# Seeding and Establishment of *Legionella pneumophila* in Hospitals: Implications for Genomic Investigations of Nosocomial Legionnaires’ Disease

**DOI:** 10.1093/cid/cix153

**Published:** 2017-02-17

**Authors:** Sophia David, Baharak Afshar, Massimo Mentasti, Christophe Ginevra, Isabelle Podglajen, Simon R. Harris, Victoria J. Chalker, Sophie Jarraud, Timothy G. Harrison, Julian Parkhill

**Affiliations:** 1 Pathogen Genomics, Wellcome Trust Sanger Institute, Cambridge, and; 2 Respiratory and Vaccine Preventable Bacteria Reference Unit, Public Health England, London, United Kingdom;; 3 European Programme for Public Health Microbiology Training, European Centre for Disease Prevention and Control, Stockholm, Sweden; and; 4 French National Reference Center of Legionella, Hospices Civils de Lyon,; 5 International Center of Infectiology Research, INSERM, U1111, CNRS, UMR5308, Université Lyon 1, École Normale Supérieure de Lyon, and; 6 Microbiology, Assistance publique–Hôpitaux de Paris, Hôpital Européen Georges Pompidou, Paris, France

**Keywords:** whole-genome sequencing, bacterial genomics, Legionnaires’ disease, *Legionella pneumophila*, nosocomial infections.

## Abstract

**Background.:**

Legionnaires’ disease is an important cause of hospital-acquired pneumonia and is caused by infection with the bacterium *Legionella*. Because current typing methods often fail to resolve the infection source in possible nosocomial cases, we aimed to determine whether whole-genome sequencing (WGS) could be used to support or refute suspected links between cases and hospitals. We focused on cases involving a major nosocomial-associated strain, *L. pneumophila* sequence type (ST) 1.

**Methods.:**

WGS data from 229 *L. pneumophila* ST1 isolates were analyzed, including 99 isolates from the water systems of 17 hospitals and 42 clinical isolates from patients with confirmed or suspected hospital-acquired infections, as well as isolates obtained from or associated with community-acquired sources of Legionnaires’ disease.

**Results.:**

Phylogenetic analysis demonstrated that all hospitals from which multiple isolates were obtained have been colonized by 1 or more distinct ST1 populations. However, deep sampling of 1 hospital also revealed the existence of substantial diversity and ward-specific microevolution within the population. Across all hospitals, suspected links with cases were supported with WGS, although the degree of support was dependent on the depth of environmental sampling and available contextual information. Finally, phylogeographic analysis revealed that hospitals have been seeded with *L. pneumophila* via both local and international spread of ST1.

**Conclusions.:**

WGS can be used to support or refute suspected links between hospitals and Legionnaires’ disease cases. However, deep hospital sampling is frequently required due to the potential coexistence of multiple populations, existence of substantial diversity, and similarity of hospital isolates to local populations.

## Summary

Whole-genome sequencing can be used to support or refute suspected links between hospital water systems and Legionnaires’ disease cases. However, caveats regarding the interpretation of genomic data from *Legionella pneumophila* are described that should be considered in future investigations.

**(See the Editorial Commentary by Beatson and Bartley on pages 1260-2.)**

*Legionella* is a genus of gram-negative bacteria that comprises species found in natural aquatic and soil habitats [1]. It also now colonizes modern, man-made water systems from which humans can become infected. Infection can result in a severe, life-threatening pneumonia called Legionnaires’ disease (LD). While most cases are community-acquired, LD is also an important cause of hospital-acquired pneumonia [2]. Most nosocomial cases are linked to inhalation or aspiration of contaminated drinking water [3], although sources such as decorative fountains, humidifiers, and cooling towers have also been implicated [4–7]. More than 90% of LD cases are caused by *Legionella pneumophila* [8].

There have been several reports of long-term colonization of hospital water systems with *L. pneumophila*, often with persistence of the same strain [9–12]. In particular, sequence type (ST) 1, as defined by the gold standard typing method, sequence-based typing (analogous to multilocus sequence typing) [13–16], has been shown to colonize several hospitals worldwide and has often been implicated as the cause of nosocomial LD [12, 17, 18]. However, as ST1 isolates are detected commonly in environmental sources, both within hospitals and elsewhere [17, 19, 20], the source of infection in possible nosocomial cases is often unresolved with sequence-based typing.

Here, we used whole-genome sequencing (WGS) to examine suspected links between multiple hospital water systems and LD cases caused by *L. pneumophila* ST1. In particular, we performed a detailed investigation of 7 cases associated with an anonymous hospital, hospital A (Essex, United Kingdom). Deep environmental sampling of this hospital allowed comparison with another previously studied and deeply sampled hospital, the Wesley Hospital (hospital B, Queensland, Australia), that was found to be colonized by a single, although surprisingly diverse, population of ST1 using WGS (although the study did not describe the strain as ST1) [21]. We also aimed to understand the evolutionary context and similarity of hospital populations within the global ST1 phylogeny and, finally, to assess the implications of these results for future WGS-based investigations of nosocomial-associated infections.

## METHODS

### Bacterial Isolates

WGS data from an internationally sampled collection of 229 ST1 or ST1-derived isolates were used in this study (Supplementary Table 1). Complete or draft genomes for 138 isolates have been previously published, whereas 91 isolates are newly sequenced. The collection includes 99 environmental isolates from the water systems of 17 hospitals spanning 5 countries (United Kingdom, France, Spain, Denmark, and Australia). Multiple environmental isolates were obtained from 5 of these hospitals (hospital A, n = 38; hospital B, n = 39; hospital C, n = 5; hospital D, n = 3; hospital E, n = 2), while a single environmental isolate was obtained from 12 hospitals. Forty-two clinical isolates from patients with confirmed or suspected links to 20 different hospitals, including 10 hospitals from which we also obtained 1 or more environmental isolates, were included. Of the remaining 88 isolates in the collection, 47 are from or associated with community-acquired sources of LD, and 3 were sampled from a cruise ship, while the sampling context of 38 isolates is unknown.

### Culture and DNA Extraction

*Legionella pneumophila* isolates, stored at −80°C, were grown at 37°C on buffered charcoal-yeast extract agar for 48–72 hours prior to DNA extraction. High-quality DNA was extracted using the Wizard or Maxwell LEV Blood DNA kit (Promega UK, Southampton, United Kingdom) according to the manufacturer’s instructions. DNA was eluted in 1× TE buffer pH 8.0 and quantified using GloMax (Promega UK).

### Whole-Genome Sequencing

Isolates were sequenced by the core sequencing facilities at Public Health England using the Illumina HiSeq platform with 100-bp paired-end reads, or at the Wellcome Trust Sanger Institute using the Illumina MiSeq platform with 150-bp paired-end reads. Library construction was performed as described previously [22, 23]. Raw reads for all newly sequenced isolates were deposited in the European Nucleotide Archive (study accession numbers ERP003631 and ERP015468).

### Mapping of Sequence Reads and Phylogenetic Analyses

Sequence reads were mapped to the Paris genome [24] using SMALT software version 0.7.4 (http://www.sanger.ac.uk/science/tools/smalt-0). Bases were called using an in-house pipeline comprising SAMtools [25], mpileup, and BCFtools, as described previously [26]. Recombined regions were identified and removed from the alignment using Gubbins software [27]. A maximum likelihood tree was generated using variable sites that remained after recombination removal using RAxML version 7.0.3 [28]. Among-site rate variation was accounted for using a γ correction, and 100 random bootstrap replicates were performed to analyze support for nodes.

## RESULTS

### Hospital Populations Comprise Distinct Lineages of *L. pneumophila* ST1

**Figure 1.**
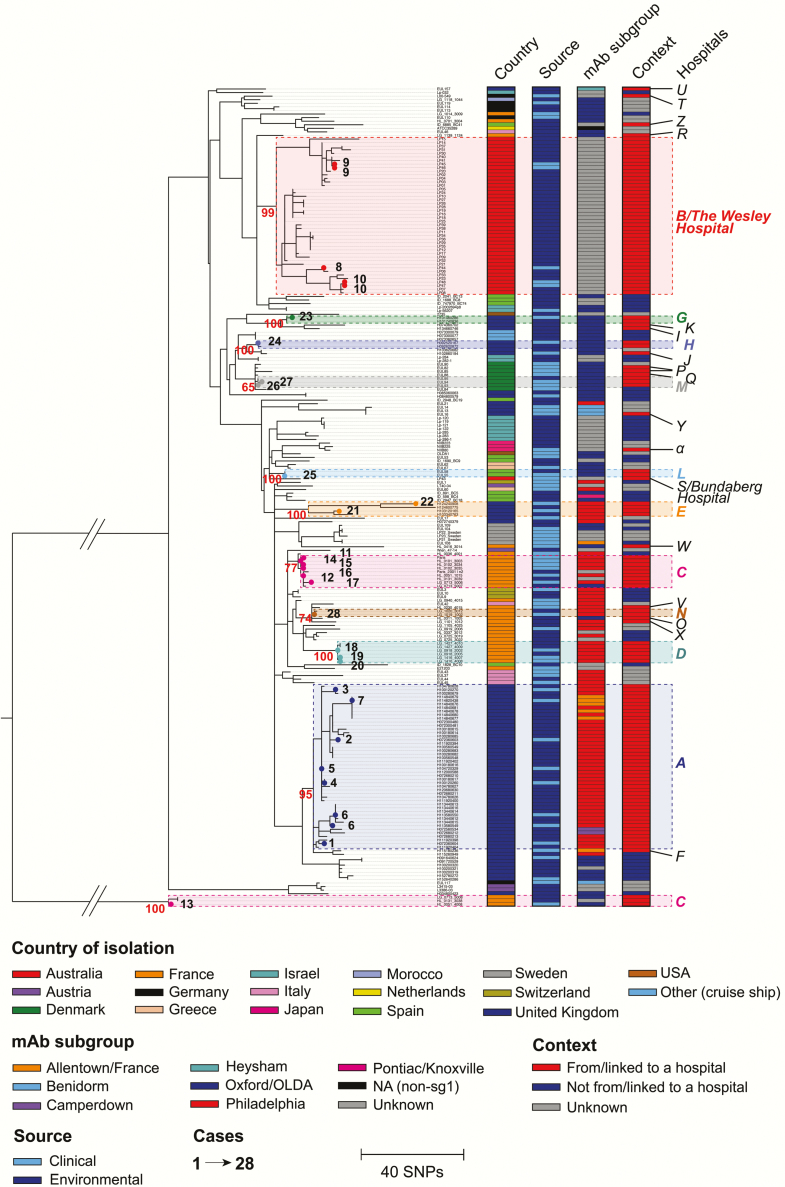
A maximum likelihood tree of 229 sequence type (ST) 1 and ST1-derived isolates constructed using 1741 single-nucleotide polymorphisms (SNPs) identified after the removal of recombined regions. Environmental isolates from and clinical isolates linked to 27 different hospitals are included. Isolates from or potentially linked to the water systems of 10 of these hospitals (from which at least 1 environmental isolate and 1 clinical isolate was obtained) are colored within the tree itself. Clinical isolates from 28 suspected cases linked to these 10 hospitals are indicated by small circles (colored according to the hospital) and numbered within the tree. Clinical isolates obtained from the same patient have the same number. Bootstrap values obtained for nodes from which isolates from the 10 hospitals are descended are shown in red. The broken branches have been reduced to a third of their original size. The scale shows the number of SNPs. Abbreviations: mAb, monoclonal antibody; NA, not applicable; OLDA, ;SNP, single-nucleotide polymorphism; USA, United States.

We investigated the phylogenetic context of 99 environmental isolates sampled from the water systems of 17 hospitals, together with 42 clinical isolates from LD patients with confirmed or suspected hospital-acquired infections, within an internationally sampled collection of 229 *L. pneumophila* ST1 or ST1-derived genomes (Supplementary Table 1). To construct a phylogenetic tree, sequence reads were mapped to the complete genome of the Paris strain (an ST1) [24], and a total of 62395 single-nucleotide polymorphisms (SNPs) were identified among all isolates. Because recombination accounts for a large proportion of diversity within the ST1 lineage [29], Gubbins software was used to identify and remove recombined regions from the alignment. A total of 382 putative recombined regions, containing 97.2% of the total SNPs (but affecting a mean of just 5.1% of each genome [range, 0.85%–14.5%]), was identified and removed (Supplementary Table 2). The remaining 1741 SNPs were used to construct a phylogenetic tree (Figure 1). SNP differences between isolates that are provided from here on represent only SNPs that have arisen via de novo mutation and exclude those in recombined regions, unless stated otherwise.

Using the phylogenetic tree, we first investigated whether 5 hospitals (hospitals A–E) from which multiple ST1 isolates were obtained have been colonized by distinct or mixed ST1 populations. Figure 1 shows that environmental isolates sampled from the water system of 4 hospitals (A, B, D, and E) indeed cluster together, demonstrating the existence of single ST1 populations (although only small numbers were obtained from hospitals D [n = 3] and E [n = 2]). Interestingly though, environmental isolates from hospital C form 2 distinct clusters, differing by up to 300 SNPs. Nevertheless, the finding that all 5 hospitals have been colonized by a limited number of distinct ST1 populations is an important prerequisite for using WGS in case investigations.

### WGS Can Be Used to Support or Refute Links Between LD Cases and Hospitals

**Figure 2.**
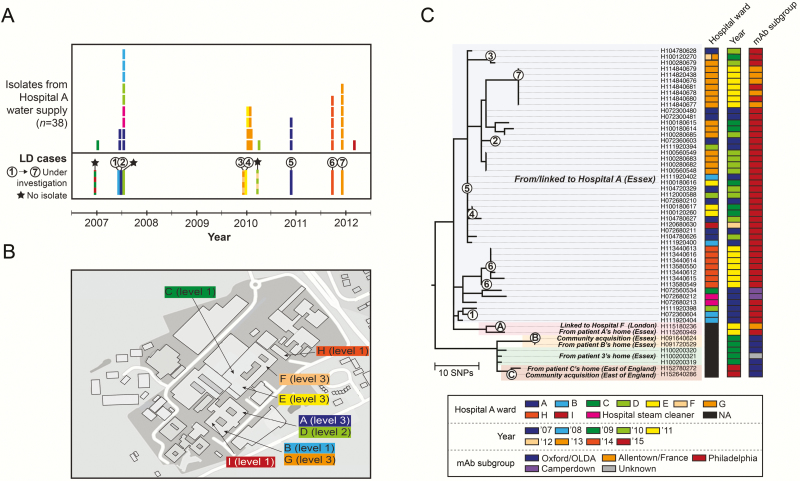
*A*, The time frame in which 10 cases of Legionnaires’ disease that are considered to have been acquired in hospital A occurred between the end of 2006 and 2011 (bottom panel). Clinical isolates were obtained from 7 of these cases, as indicated. Environmental isolates were also obtained between 2007 and 2012 from the hospital water supply, usually after each Legionnaires’ disease incident (top panel). Isolates are colored according to the hospital ward(s) in which the patient stayed (clinical isolates) or they were sampled (environmental isolates). *B*, A plan of hospital A, showing the wards in which the patients stayed, and those in which the environmental isolates were obtained. *C*, A zoomed-in section of the maximum likelihood tree presented in Figure 1, showing environmental isolates from and clinical isolates linked to hospital A. Clinical isolates from 7 cases linked to hospital A are indicated by small circles and numbered 1–7 (2 isolates were obtained from case 6). Closely related isolates sampled from nearby homes are also shown, including the home of a patient (case 3) who spent part of their incubation period in hospital A as well as 3 homes of patients who had no known epidemiological link to hospital A. Clinical isolates from these latter 3 patients are indicated by small circles and labeled A–C. Clinical isolate A was obtained from a patient whose incubation period was spent both at home and in hospital F, while isolates B and C are from patients with no known epidemiological links to any hospitals. Bootstrap values for all nodes in the tree are provided in Supplementary Figure 1. Abbreviations: LD, Legionnaires’ disease; mAb, monoclonal antibody; NA, not applicable; SNP, single-nucleotide polymorphism.

We next investigated whether the WGS data support confirmed or suspected links between hospital water systems and LD cases. In particular, we performed a detailed examination of 7 cases that occurred between 2007 and 2011, all of which are considered to have been acquired from hospital A (Table 1). All clinical isolates, except 1 obtained from the most recent case (November 2011) were typed as ST1, monoclonal antibody (mAb) subgroup Philadelphia, an uncommon strain in England [19], but also the assigned type of the hospital isolates. Meanwhile, the clinical isolate from the most recent case was typed as ST1, mAb subgroup Allentown/France, also matching the type of more recently sampled hospital isolates from 2011. Here, we compared 8 clinical isolates from these cases (2 of which come from 1 patient) with 38 environmental isolates from hospital A, within the context of our large collection of sequenced ST1/ST1-derived isolates. Importantly, the collection includes contemporary isolates from or associated with another 7 hospitals (hospitals E–K) and community-acquired sources in the local area of London/east of England. Phylogenetic analyses show that all 8 clinical isolates are nested within, and thus derived from, the clade of isolates sampled from hospital A (Figures 1 and 2 and Supplementary Figure 1). This finding provides strong evidence that the infections were indeed acquired from the hospital (Table 1). Furthermore, each of the clinical isolates differ by just 0–4 SNPs from the closest hospital isolate, providing further supporting evidence. Further analysis (see Supplementary Materials) found that the number of hospital A isolates needed to have been analyzed to show that each clinical isolate is derived from the hospital clade in >90% of combinations ranges from 3 to 15, depending on the clinical isolate used (Supplementary Figure 2).

**Table 1.** Genomic Evidence to Support 28 Suspected Links Between Hospital Water Systems and Legionnaires’ Disease Cases, From Which at Least 1 Hospital Isolate and 1 Clinical Isolate Was Obtained and Analyzed Using Whole-Genome Sequencing

Suspected Hospital	Date of Incident	Known Exposures During the Incubation Period (~18 d Prior to Onset of Symptoms)	Clinical Isolate(s)	Does the Clinical Isolate Cluster Most Closely With a Hospital Water Isolate? (No. of SNPs)^a^	Genomic Evidence^b^
Hospital A, Essex, UK	May 2007	Hospital A (11–18 d), home	H072360604 (case 1)	Yes (4 SNPs)	A
	May 2007	Hospital A (~12 d)	H072360603 (case 2)	Yes (3 SNPs)	A
	December 2009	Hospital A (~4 d), home and local area	H100120270 (case 3)	Yes (1 SNP)	A
	December 2009	Hospital A (~7 d), home and local area	H100120260 (case 4)	Yes (0 SNPs)	A
	November 2010	Hospital A (~7 d), home and local area	H104720329 (case 5)	Yes (0 SNPs)	A
	August 2011	Hospital A (at least 10 d)	H113580549, H113580550 (case 6)	Yes (3 and 0 SNPs, respectively)	A
	November 2011	Hospital A (at least 10 d)	H114820438 (case 7)	Yes (0 SNPs)	A
Hospital B, Wesley Hospital, Queensland, Australia	October 2011	Hospital B	LP44 (case 8)	Yes (1 SNP)	A
	May 2013	Hospital B only	LP45, LP46 (case 9)	Yes (both 1 SNP)	A
	June 2013	Hospital B only	LP47 and LP48 (case 10)	Yes (1 and 2 SNPs, respectively)	A
Hospital C, Paris, France	March 2002	Hospital C (13 d) and another hospital near Paris (5 d)	Paris (case 11)	Yes (2 SNPs different to isolate from hospital C). No isolates obtained from the other hospital	C (acquisition from other hospital cannot be excluded)
	December 2000	Hospital C only	HL 0051 1015 (case 12)	Yes (0 SNPs)	C
	December 2000	Hospital C (~17 d)	HL 0051 4008 (case 13)	Yes (4 SNPs)	C
	December 2000	Hospital C (~12 d)	HL 0101 3003 (case 14)	Yes (1 SNP)	C
	December 2000	Hospital C (~4 d), home	HL 0102 3034 (case 15)	Yes (2 SNPs)	C
	December 2000	Hospital C (~4 d), home	HL 0102 3035 (case 16)	Yes (2 SNPs)	C
	March 2007	Hospital C only	LG 0713 5006 (case 17)	Yes (3 SNPs)	A
Hospital D, near Marseille, France	April 2009	Hospital D (~4 d), home	LG 0918 2002 (case 18)	Yes (0 SNPs)	C
	April 2014	Hospital D (~3 d), home (~3 d)	LG 1416 4007 (case 19)	Yes (1 SNP)	A
	April 2014	Hospital D (~5 d)	LG 1416 4008 (case 20)	Yes (1 SNP)	A
Hospital E, London, UK	June 2010	Hospital E (at least 10 d)	H103120165 (case 21)	Yes (7 SNPs)	B
	October 2012	Hospital E (<10 d)	H124240908 (case 22)	Yes (33 SNPs)	B
Hospital G, Cambridgeshire, UK	April 2010	Hospital G (<10 d)	H101460286 (case 23)	Yes (2 SNPs)	D
Hospital H, London, UK	June 2009	Hospital H (at least 10 d)	H092520167 (case 24)	Yes (1 SNP)	D
Hospital L, Cáceres Province, Spain	April 1994	Hospital L	EUL 55 (case 25)	Yes (0 SNPs)	D
Hospital M, Copenhagen, Denmark	October 1992	Hospital M only	EUL 93 (case 26)	Yes (0 SNPs)	D
	December 1992	Hospital M only	EUL 94 (case 27)	Yes (1 SNP)	D
Hospital N, near Marseille, France	April 2010	Hospital N only	LG 1019 1002 (case 28)	Yes (1 SNP)	D

Abbreviation: SNP, single-nucleotide polymorphism.

^a^SNP differences refer to those that remain after the removal of recombined regions.

^b^Different types of genomic evidence were categorized. A: The clinical isolate(s) is derived from the most recent common ancestor (MRCA) of the hospital isolates, and differs by <5 SNPs to the closest hospital water isolate. Strong evidence that the infection was hospital-acquired. B: The clinical isolate(s) is derived from the MRCA of the hospital isolates, but differs by >5 SNPs to the closest hospital water isolate. Good evidence that the infection was hospital-acquired. C: The clinical isolate(s) clusters most closely with hospital isolates, and is <5 SNPs different from the closest hospital isolate. However, the clinical isolate(s) is not derived from the MRCA of the sampled hospital isolates. Acquisition from elsewhere cannot be excluded on the basis of genomic evidence alone. D: The clinical isolate(s) clusters most closely to and differs by <5 SNPs from the hospital isolate. However, the recovery of only 1 hospital isolate prevents the determination of whether the clinical isolate is derived from hospital isolates. Acquisition from elsewhere cannot be excluded on the basis of genomic evidence alone.

Interestingly, some isolates from or associated with community sources in the local area, as well as a clinical isolate from a patient who spent part of their incubation period in hospital F (London, United Kingdom), also cluster closely with hospital A isolates (Figure 2). These include 3 isolates (H100200319, H100200320, H100200321) obtained from a patient’s home (case 3) who spent their incubation period both at home and in hospital A. The investigation at the time ruled out the home as a potential source based on mAb subtyping results, and this finding is now also supported by WGS data.

**Figure 3.**
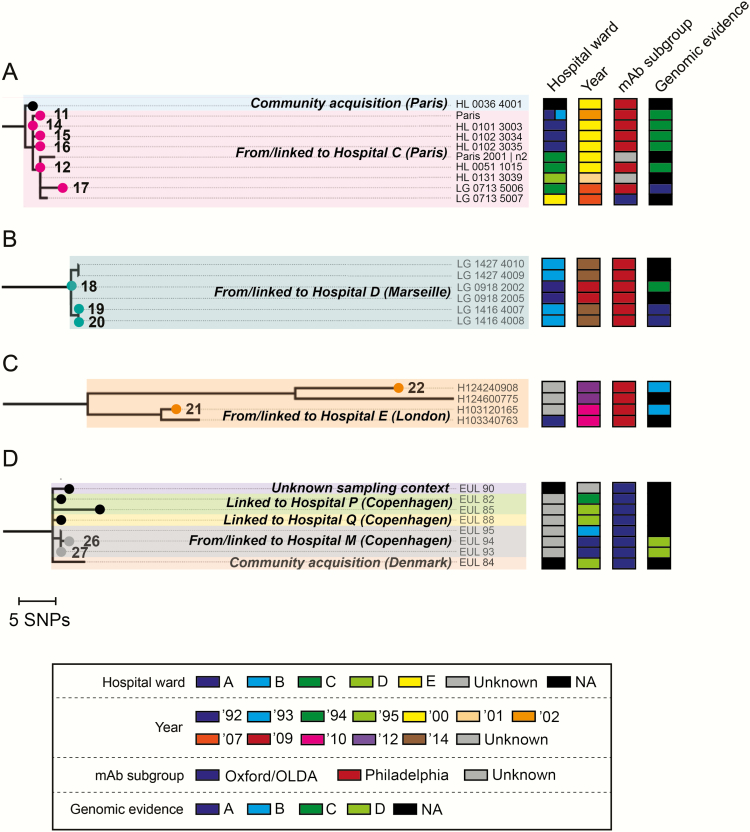
*A–D*, Zoomed-in sections of the maximum likelihood tree presented in Figure 1. All clinical isolates are indicated by small circles, with those from the 28 cases under investigation colored and numbered as in Figure 1. Where applicable, isolates are additionally colored in the right-hand panel according to the hospital ward(s) in which the patient stayed (clinical isolates) or they were sampled (environmental isolates). Clinical isolates from the 28 cases under investigation are also colored in the right-hand panel by the strength of genomic evidence for hospital acquisition (see Table 1). Abbreviations: mAb, monoclonal antibody; NA, not applicable; SNP, single-nucleotide polymorphism.

Examination of other suspected links between cases and hospitals further demonstrates how the interpretation and strength of evidence obtained is highly dependent on both sampling and contextual information (Table 1 and Figure 3). For example, phylogenetic analyses confirmed previous findings [21] that 3 cases associated with hospital B were acquired within the hospital since the clinical isolates are nested within, and thus derived from, the hospital clade and differ by just 1–2 SNPs from the closest hospital isolate (Figure 1 and Table 1). As with hospital A, the large number of hospital isolates analyzed facilitated these findings. Furthermore, investigation of 2 cases associated with hospital E (from 2010 and 2012) revealed that whereas the 2 clinical isolates each cluster most closely with a single contemporary hospital isolate, they differ by 7 and 33 SNPs, respectively, to these hospital isolates. If each pair (comprising 1 clinical and 1 contemporary environmental isolate) were analyzed alone, an investigation might refute a link between the second case and the hospital due to the high SNP differences. However, phylogenetic analysis of both pairs within the large ST1 collection shows that the 4 isolates cluster together and that both clinical isolates are derived from the most recent common ancestor of the 2 hospital isolates (which presumably was a hospital isolate itself unless the hospital has been seeded multiple times). This provides good evidence to support hospital acquisition of both infections. On the other hand, we investigated several links between cases where only 1 environmental isolate from the suspected hospital was obtained (eg, hospitals G, H, L, M, and N) (Table 1). In all such cases, the clinical isolates are more closely related to the environmental isolate from each suspected hospital than from anywhere else, differing by just 0–2 SNPs. However, when only 1 environmental isolate is obtained, it is impossible to determine whether the clinical isolate is derived from hospital isolates, even if the isolates are very similar or even identical, due to the existence of highly similar and identical *L. pneumophila* isolates in multiple sources [29]. This means that acquisition from elsewhere cannot be excluded, except based on epidemiological information.

### Substantial Diversity Within Single Hospital Populations

Despite colonization of several hospitals with distinct ST1 populations, it is clear from both the previous study by Bartley et al [21] and our genomic analyses that considerable diversity exists within at least some lineages. For example, 1682 SNPs were found among the 38 hospital A isolates and 891 SNPs among the 39 hospital B isolates before recombination removal. However, once recombined regions were removed, a total of 72 and 145 SNPs remained in the 2 hospital lineages with maximum pairwise differences of 25 and 44 SNPs, respectively. Further details of recombination events within hospital A are provided in the Supplementary Materials. We also detected variation in mAb subtypes within the hospital A population (Figure 2), and genomic comparisons allowed us to predict the genetic basis of switching events (see Supplementary Materials).

### Evidence for Local Microevolution Within Hospital Populations

Given the substantial level of diversity observed among hospital A isolates, we explored whether isolates clustered by hospital ward, as was shown in hospital B [21]. Figure 2 shows that there is some clustering by ward and that 7 of the 8 clinical isolates are most similar to 1 or more contemporary hospital isolates obtained from the same ward in which the patient stayed. Further details are provided in the Supplementary Materials.

### Long-term Stability of Hospital Strains

Despite the discovery of substantial diversity within hospital populations, we also observed long-term persistence of highly similar and even identical strains within several hospitals (see Supplementary Materials). For example, isolates with no SNPs were sampled from hospital A over 6 years (2007–2012).

### Evidence for Hospital Seeding via Local and International Spread of ST1

Phylogeographic analysis of the 229 ST1/ST1-derived isolates revealed many examples whereby isolates cluster with epidemiologically unrelated isolates from the same region (Figure 1). In addition to isolates from hospital A and the surrounding area, another notable example is 6 isolates sampled from or associated with 3 different hospitals in the greater Copenhagen area (hospitals M, P, and Q), which are no more than 10 km apart, that differ by 2–8 SNPs (not including differences between isolates from the same hospital) (Figure 3). Intriguingly, there are also isolates from distant countries that differ by very few SNPs. Just 14 SNPs were identified between an environmental isolate (LG 1139 1124) from hospital R (France) in 2011 and an environmental isolate (LP25) from hospital B (Australia) in 2013, although 1082 SNPs are found if 5 recombined regions that differentiate the isolates are included.

## DISCUSSION

Although the possibility of using WGS in community-acquired LD investigations has been well explored [30–34], its potential role in resolving nosocomial-associated investigations has been less studied [21, 35, 36]. Using WGS data from 229 *L. pneumophila* isolates belonging (or closely related) to a major nosocomial-associated strain, ST1, this study has demonstrated the unparalleled resolution of WGS and its improved capability to trace source acquisition over current methods. It has also shown occasional incongruences between mAb subtyping results and epidemiological information (eg, hospital A isolates, some identical by SNPs, have different mAb subtypes), which may have implications for the use of mAb typing for epidemiological investigations. However, the study has also highlighted several limitations faced in WGS-based investigations of *L. pneumophila*, attributable to its unusual biology and evolution, which should be considered in the future interpretations of genomic data.

The first caveat is related to the finding from this study and others [37, 38], that epidemiologically unrelated isolates exist that are highly similar at the SNP level. The implication is that although a low number of SNPs between isolates supports a link, it does not provide absolute evidence of one. Therefore, in the several suspected nosocomial cases from which only 1 clinical isolate was obtained and compared with just 1 hospital isolate, we were unable to rule out acquisition from elsewhere on the basis of the genomic data alone. However, stronger genomic evidence of a link can come from the observation that a clinical isolate is nested within and thus derived from a clade of hospital isolates. Such evidence can be achieved only by obtaining multiple hospital isolates and, for example, was successfully used to confirm suspected links to hospital A and, previously, to hospital B [21]. However, even recovery of multiple isolates (especially in low numbers) does not guarantee obtaining this key piece of supporting evidence, as with cases linked to hospitals C and D. Further work is required to determine a robust recommendation of the number of hospital isolates required (both for ST1 and other STs), but certainly a minimum of 3 is needed, with the likelihood of achieving good evidence to support a link increasing for each additional isolate analyzed. These findings also reinforce the importance of including contextual phylogenetic information from the same ST, and of combining epidemiological and typing data.

The requirement for obtaining multiple environmental samples is also reinforced by our discovery that pairwise SNP differences between isolates from the same hospital can frequently outnumber those found between hospital isolates and epidemiologically unrelated isolates from sources elsewhere, particularly the local area (eg, nearby homes). Thus, without an understanding of the hospital diversity in the context of local diversity, spurious links could be made on SNP differences alone. However, further analysis of non-SNP genetic information (eg, indels, recombined regions, mobile elements) could also help improve source attribution to a limited extent, as was shown in previous analysis of the Wesley Hospital [21].

Finally, this study reinforces previous findings that the ST1 lineage has surprisingly limited diversity excluding recombined regions [29]. The discovery of highly similar ST1 isolates within nearby hospitals (and other community sources) suggests that hospitals may be seeded by the local “endemic” ST1 strain, either via the main water supply or other means. However, the finding that globally dispersed ST1 isolates also differ by few SNPs demonstrates long-distance spread, as previously reported [29], via as yet unknown mechanisms, and subsequent seeding of environmental sources including hospitals. SNP differences between isolates from distant countries are sometimes similar to or even lower than those between isolates from the same hospital (eg, hospital A), which could suggest spreading within a similar time frame to that in which hospital populations have diversified. This time frame could span years to decades considering, for example, that hospital A was opened in the 1970s, and thus cannot have been colonized more than approximately 40 years. However, this hypothesis assumes that each hospital has been seeded once, or a limited number of times, and therefore that the observed diversity has been generated completely, or mostly, within the hospital itself since the initial colonization event(s). Because isolates at least partially cluster by ward in hospital A and hospital B (the Wesley Hospital), we believe this is a safe assumption for these hospitals. Secondly, the hypothesis also assumes that the evolutionary rate of ST1 remains relatively constant, which may not be correct. It could be that it is higher in hospital water systems than in other environments due to favorable replication conditions, meaning that international dispersal need not be explained by such rapid spread. As suggested previously [29], *L. pneumophila* could also undergo dormancy periods, which would explain our observations of identical isolates sampled many years apart. Deepening our understanding of the speed and mechanisms by which *L. pneumophila* spreads locally and globally, and gaining further insights into its evolutionary rate and potential dormancy, will be important for informing future WGS-based investigations.

## Supplementary Data

Supplementary materials are available at *Clinical Infectious Diseases* online. Consisting of data provided by the author to benefit the reader, the posted materials are not copyedited and are the sole responsibility of the author, so questions or comments should be addressed to the author.

## Supplementary Material

David_2016_revised_supplementary_material

## Notes

***Acknowledgments.*** We thank the library-generation, sequencing, and informatics teams at the Wellcome Trust Sanger Institute and Public Health England for their assistance. We also thank our Public Health and Microbiology colleagues for epidemiological data and submission of specimens and isolates, as well as the staff within the Respiratory and Vaccine Preventable Bacteria Reference Unit at Public Health England for isolation, identification, and discrimination of *Legionella*.

***Disclaimer.*** The funder had no role in study design, data collection and interpretation, or the decision to submit the work for publication.

***Financial support.*** This work was supported by the Wellcome Trust (grant number 098051).

***Potential conflicts of interest.*** J. P. has received funding for conference travel and accommodation from Illumina Inc. All other authors report no potential conflicts. All authors have submitted the ICMJE Form for Disclosure of Potential Conflicts of Interest. Conflicts that the editors consider relevant to the content of the manuscript have been disclosed.
