# Supplementary material for: Seeding and Establishment of Legionella pneumophila in Hospitals: Implications for Genomic Investigations of Nosocomial Legionnaires’ Disease
Source: Clin Infect Dis. 2017 Feb 17;64(9):1251–9. doi: 10.1093/cid/cix153 (PMC5399934; doi:10.1093/cid/cix153)

**Supplementary Appendix**

**Seeding and establishment of *Legionella pneumophila* in hospitals; implications for genomic investigations of nosocomial Legionnaires’ disease**

Sophia David^1,2^, Baharak Afshar^2,3^, Massimo Mentasti^2^, Christophe Ginevra^4,5^, Isabelle Podglajen^6^, Simon R. Harris^1^, Victoria J. Chalker^2^, Sophie Jarraud^4,5^, Timothy G. Harrison^2^ & Julian Parkhill^1^

^1^Pathogen Genomics, Wellcome Trust Sanger Institute, Cambridge, United Kingdom; ^2^Respiratory and Vaccine Preventable Bacteria Reference Unit, Public Health England, London, United Kingdom; ^3^The European Programme for Public Health Microbiology Training (EUPHEM), ECDC, Stockholm, Sweden; ^4^French National Reference Center of *Legionella*, Hospices Civils de Lyon, France; ^5^International Center of Infectiology Research, Inserm, U1111, CNRS, UMR5308, Université Lyon 1, École Normale Supérieure de Lyon, Lyon, F-69008, France; ^6^Microbiology, AP-HP, Hôpital Européen Georges Pompidou, Paris, France

**Contents**

Supplementary Methods…………....………….…………………………………………………………………….Page 2

Supplementary Results…………….………………………………………………………………………...……….Page 2

Supplementary Table 1 (Table S1)……..………………………………………………………………………..Page 5

Supplementary Table 2 (Table S2)……..……………………………………………………………….……..Page 20

Supplementary Figure 1 (Figure S1)……………………………………………….…………………………Page 40

Supplementary Figure 2 (Figure S2)……………………………………………….…………………………Page 41

**Supplementary Methods**

***Environmental sampling analysis***

Subsets containing between one and 38 environmental isolates from Hospital A were created by sampling all possible combinations of the 38 environmental isolates up to 100,000 for each subset size. The proportion of times that each of the eight clinical isolates associated with Hospital A was found to be derived from the subset of environmental isolates in the phylogenetic tree was calculated for each subset size (not including occasions where the clinical isolate is identical to the most recent common ancestor (MRCA) of the environmental isolates).

**Supplementary Results**

***Environmental sampling analysis***

To retrospectively investigate how many environmental samples would have been needed to provide good support of hospital acquisition for each of the seven cases associated with Hospital A, we analysed each clinical isolate with different numbers of environmental isolates. For five cases, we found that comparison of three hospital isolates together with each clinical isolate would have been sufficient to demonstrate that the clinical isolate is derived from the hospital clade, given ≥90% of the possible combinations of three isolates (**Figure S2**). However, for all sampled combinations of a given subset size to provide good support of hospital acquisition for the five cases, 5-6 hospital isolates were required. Fifteen environmental isolates would have been necessary for the remaining two cases, given ≥90% of the sampled combinations, regardless of which of the two clinical isolates was used for case 6 (**Figure S2**). Yet for all combinations of a given subset size to provide good support of hospital acquisition, up to 29 hospital isolates were needed for both cases.

***Recombination amongst Hospital A isolates***

Gubbins detected the occurrence of seven putative recombination events within the Hospital A lineage (of which two are just 6bp and 41bp and likely the result of sequencing or mapping artefacts), which, once removed, leaves a total of 72 SNPs between the 38 environmental isolates and a maximum difference of 25 SNPs between any pair. Interestingly, the five larger recombination regions (ranging in size from 1,442bp to 38,021bp) all occurred on the same branch of the phylogenetic tree, affecting the isolates, H072560534 and H072680212, and thus may have been acquired on the same occasion.

That recombination events were detected within the ST1 populations of both Hospitals A and B also indicates the existence of other (probable non-ST1) *L. pneumophila* strains within each hospital water supply, assuming that the hospital populations have been restricted to the hospital water system and that the hospitals have not been re-seeded with newly recombined strains.

***Variation in monoclonal antibody (mAb) subtypes within Hospital A isolates***

Overall, 32 of 38 environmental isolates from Hospital A and seven of the eight associated clinical isolates belong to the mAb subtype, Philadelphia (**Figure 2**). However, two closely related environmental isolates sampled from the hospital water supply in 2007, which are the same two isolates affected by the five recombination events, were typed as Camperdown. The genetic determinants of the mAb subtypes are not well understood but are presumably located within the lipopolysaccharide (LPS) locus. Thus, we predict that one of the recombination events that spans the LPS locus, ranging from 923,274bp (*lpp0825*) to 931,183bp (*lpp0831*) with respect to the Paris reference genome, and which introduces a total of 107 SNPs, is the cause of the mAb switch. Intriguingly, the one clinical isolate and four environmental isolates sampled in 2011 and characterised as mAb subtype, Allentown/France, cluster together in the phylogenetic tree along with two isolates typed as Philadelphia (**Figure 2**). No SNPs were identified between all seven isolates, both before and after the removal of recombined regions. We thus searched for other differences that could explain the differing mAb subtypes including insertions, deletions and differences in gene content. The only observed difference affecting the LPS locus was a single insertion of a thymine base at 935,649 (which cases a frameshift about 80% through *lpp0835*) in the five Allentown/France isolates, but not the two Philadelphia isolates, which we predict changes the mAb subtype.

***Evidence of ward-specific evolution***

**Figure 2** shows that there is some clustering of isolates by ward in Hospital A and that seven of the eight clinical isolates are most similar to one or more contemporary environmental isolates sampled from the same ward in which the patient was a resident. For example, all five environmental isolates sampled from various outlets in ward H in 2011 cluster together, differing by 0-4 SNPs, and also cluster with two clinical isolates (H113580549, H113580550) obtained from the post-mortem lung tissue of a patient (case 6) who stayed in the same ward. Another example is the clinical isolate, H100120260, obtained from a patient (case 4) who stayed in ward E, which has no SNP differences with an environmental isolate, H100180617, sampled from a shower in the same ward. The one clinical isolate (H072360603) that is not most similar to an environmental isolate from the same ward in which the patient (case 2) stayed (ward A) nevertheless differs by just 4 SNPs from contemporary isolates from the same ward (H072300480 and H072300481).

We also found some putative evidence of ward-specific evolution in Hospital C (**Figure 3**). For example, four clinical isolates (Paris, HL 0101 3003, HL 0102 3034 and HL 0102 3035) obtained from patients who were treated in the intensive care unit (cardiac surgery) cluster together while one environmental isolate (Paris 2001 I n2) obtained from the nephrology ward also clusters closely with two clinical isolates (HL 0051 1015 and LG 0713 5006) from patients who were treated in this ward. Furthermore, our phylogenetic analyses show that both ST1 populations detected within this hospital have co-existed within the same wards.

***Long-term stability of hospital strains***

Long-term persistence was also evident in Hospital C where, for example, two environmental isolates (HL 0131 3038 and LG 0713 5008) with no SNPs were sampled more than five years apart, and in Hospital D where environmental isolates sampled in 2009 and 2014 differ by just 1 SNP.

**Table S1.** 229 ST1 and ST1-derived isolates used in the study. ST1-derived isolates refer to those that have been previously shown to be closely related to, and to be evolved from, ST1 isolates (David *et al*. 2016). References are provided for previously published genomes and run accession numbers are provided for newly sequenced genomes. ST – sequence type; mAb subgroup – monoclonal antibody subgroup; Phil. – Philadelphia; All./France – Allentown/France; Camp. – Camperdown; NA – not applicable.

| **Hospital** | **Isolate** | **Source** | **Known exposures during incubation period (up to ~18 days)** | **Hospital ward (if known)** | **Date of isolation** | **Town/**  **Region** | **Country** | **ST/mAb subgroup** | **Accession number/**  **Reference** |
| --- | --- | --- | --- | --- | --- | --- | --- | --- | --- |
| ***Environmental isolates from hospitals or clinical isolates with confirmed/suspected links to hospitals (n=141)*** | | | | | | | | | |
| A | H072360604 (case 1) | Clinical (pleural fluid) | Hospital A (11-18 days), home | B | 24/05/2007 | Essex | UK | 1/Phil. | ERR1399547 |
|  | H072360603 (case 2) | Clinical (sputum) | Hospital A (~12 days) | A | 27/05/2007 | Essex | UK | 1/Phil. | ERR1399550 |
|  | H100120270 (case 3) | Clinical (sputum) | Hospital A (~4 days), home and local area | F & G | 29/12/2009 | Essex | UK | 1/Phil. | ERR1399506 |
|  | H100120260 (case 4) | Clinical (sputum) | Hospital A (~7 days), home and local area | E | 29/12/2009 | Essex | UK | 1/Phil. | ERR1399540 |
|  | H104720329 (case 5) | Clinical (sputum) | Hospital A (~7 days), home and local area | A | 19/11/2010 | Essex | UK | 1/Phil. | ERR1399526 |
|  | H113580549 (case 6) | Clinical (post-mortem sample from left lung) | Hospital A (at least 10 days) | H | 23/08/2011 | Essex | UK | 1/Phil. | ERR1399560 |
|  | H113580550 (case 6) | Clinical (post-mortem sample of right lung of same patient as above) |  | H | 23/08/2011 | Essex | UK | 1/Phil. | ERR1399535 |
|  | H114820438 (case 7) | Clinical | Hospital A (at least 10 days) | G | 24/11/2011 | Essex | UK | 1/All./  France | ERR1399537 |
|  | H072560534 | Environmental (carpet cleaner reservoir) | NA | C | 09/01/2007 | Essex | UK | 1/Camp. | ERR1399501 |
|  | H072300480 | Environmental (hot sink, heat-treated sample) | NA | A | 30/05/2007 | Essex | UK | 1/Phil. | ERR1399554 |
|  | H072300481 | Environmental (hot sink, untreated sample) | NA | A | 30/05/2007 | Essex | UK | 1/Phil. | ERR1399565 |
|  | H072680210 | Environmental (day room; hot thermostatic mixing valve) | NA | A | 07/06/2007 | Essex | UK | 1/Phil. | ERR1399562 |
|  | H072680211 | Environmental (staff room; chilled cold water) | NA | A | 07/06/2007 | Essex | UK | 1/Phil. | ERR1399556 |
|  | H072680212 | Environmental (steam cleaner) | NA | Multiple | 07/06/2007 | Essex | UK | 1/Camp. | ERR1399551 |
|  | H072680213 | Environmental (steam cleaner) | NA | Multiple | 07/06/2007 | Essex | UK | 1/Phil. | ERR1399559 |
|  | H111920394 | Environmental | NA | D | 07/06/2007 | Essex | UK | 1/Phil. | ERR1399545 |
|  | H111920398 | Environmental | NA | D | 07/06/2007 | Essex | UK | 1/Phil. | ERR1399499 |
|  | H111920400 | Environmental (sink) | NA | B | 07/06/2007 | Essex | UK | 1/Phil. | ERR1399512 |
|  | H111920402 | Environmental (same as above; acid-treated sample) | NA | B | 07/06/2007 | Essex | UK | 1/Phil. | ERR1399558 |
|  | H111920404 | Environmental (wash hand basin) | NA | B | 07/06/2007 | Essex | UK | 1/Phil. | ERR1399544 |
|  | H100180614 | Environmental (sink, hot tap) | NA | G | 31/12/2009 | Essex | UK | 1/Phil. | ERR1399523 |
|  | H100180615 | Environmental (sink, hot tap) | NA | G | 31/12/2009 | Essex | UK | 1/Phil. | ERR1399508 |
|  | H100180616 | Environmental (shower) | NA | E | 31/12/2009 | Essex | UK | 1/Phil. | ERR1399511 |
|  | H100180617 | Environmental (shower) | NA | E | 31/12/2009 | Essex | UK | 1/Phil. | ERR1399549 |
|  | H100280679 | Environmental (sink, hot tap) | NA | G | 07/01/2010 | Essex | UK | 1/Phil. | ERR1399539 |
|  | H100280682 | Environmental (shower) | NA | G | 07/01/2010 | Essex | UK | 1/Phil. | ERR1399516 |
|  | H100280683 | Environmental (shower) | NA | G | 07/01/2010 | Essex | UK | 1/Phil. | ERR1399505 |
|  | H100280685 | Environmental (toilet basin) | NA | G | 07/01/2010 | Essex | UK | 1/Phil. | ERR1399503 |
|  | H100560548 | Environmental (sink, cold tap) | NA | G | 14/01/2010 | Essex | UK | 1/Phil. | ERR1399520 |
|  | H100560549 | Environmental (shower) | NA | G | 14/01/2010 | Essex | UK | 1/Phil. | ERR1399561 |
|  | H112000588 | Environmental (from patient’s room – although no clinical isolate from patient) | NA | D | 12/03/2010 | Essex | UK | 1/Phil. | ERR1399518 |
|  | H104780626 | Environmental (sink) | NA | A | 19/11/2010 | Essex | UK | 1/Phil. | ERR1399525 |
|  | H104780627 | Environmental (sink) | NA | A | 19/11/2010 | Essex | UK | 1/Phil. | ERR1399566 |
|  | H104780628 | Environmental (sink in toilet opposite patient's bed) | NA | A | 19/11/2010 | Essex | UK | 1/Phil. | ERR1399572 |
|  | H113440612 | Environmental (basin next to bed 9) | NA | H | 24/08/2011 | Essex | UK | 1/Phil. | ERR1399555 |
|  | H113440613 | Environmental (shower in room 14) | NA | H | 24/08/2011 | Essex | UK | 1/Phil. | ERR1399533 |
|  | H113440614 | Environmental (bath in room 13) | NA | H | 24/08/2011 | Essex | UK | 1/Phil. | ERR1399570 |
|  | H113440615 | Environmental (basin in side room 6) | NA | H | 24/08/2011 | Essex | UK | 1/Phil. | ERR1399536 |
|  | H113440616 | Environmental (basin in side room 6) | NA | H | 24/08/2011 | Essex | UK | 1/Phil. | ERR1399530 |
|  | H114840676 | Environmental (toilet, cold water) | NA | G | 25/11/2011 | Essex | UK | 1/All./  France | ERR1399542 |
|  | H114840677 | Environmental (toilet, hot water) | NA | G | 25/11/2011 | Essex | UK | 1/All./  France | ERR1399553 |
|  | H114840678 | Environmental (side room 13, cold water) | NA | G | 25/11/2011 | Essex | UK | 1/All./  France | ERR1399498 |
|  | H114840679 | Environmental (side room 13, hot water) | NA | G | 25/11/2011 | Essex | UK | 1/All./  France | ERR1399567 |
|  | H114840680 | Environmental (toilet) | NA | G | 25/11/2011 | Essex | UK | 1/Phil. | ERR1399522 |
|  | H114840681 | Environmental (toilet) | NA | G | 25/11/2011 | Essex | UK | 1/Phil. | ERR1399546 |
|  | H120680630 | Environmental | NA | I | 02/02/2012 | Essex | UK | 1/Phil. | ERR1399569 |
| B/The Wesley Hospital | LP01 | Environmental | NA | East Wing/  Cardiac | 29/05/2013 | Brisbane | Australia | 1/  Unknown | Bartley et al. 2016 |
|  | LP02 | Environmental | NA | East Wing/  Cardiac | 29/05/2013 | Brisbane | Australia | 1/  Unknown | Bartley et al. 2016 |
|  | LP03 | Environmental | NA | East Wing/  Cardiac | 29/05/2013 | Brisbane | Australia | 1/  Unknown | Bartley et al. 2016 |
|  | LP04 | Environmental | NA | East Wing/  Cardiac | 29/05/2013 | Brisbane | Australia | 1/  Unknown | Bartley et al. 2016 |
|  | LP05 | Environmental | NA | East Wing/  Cardiac | 29/05/2013 | Brisbane | Australia | 1/  Unknown | Bartley et al. 2016 |
|  | LP06 | Environmental | NA | Main block/  Hematology HDU | 05/06/2013 | Brisbane | Australia | 1/  Unknown | Bartley et al. 2016 |
|  | LP07 | Environmental | NA | Main block/  Hematology HDU | 05/06/2013 | Brisbane | Australia | 1/  Unknown | Bartley et al. 2016 |
|  | LP08 | Environmental | NA | Main block/  Palliative care | 05/06/2013 | Brisbane | Australia | 1/  Unknown | Bartley et al. 2016 |
|  | LP09 | Environmental | NA | Main block/  Medical centre 1 | 06/06/2013 | Brisbane | Australia | 1/  Unknown | Bartley et al. 2016 |
|  | LP10 | Environmental | NA | Main block/  Medical centre 1 | 06/06/2013 | Brisbane | Australia | 1/  Unknown | Bartley et al. 2016 |
|  | LP11 | Environmental | NA | Main block/  Rehabilitation | 06/06/2013 | Brisbane | Australia | 1/  Unknown | Bartley et al. 2016 |
|  | LP12 | Environmental | NA | Main block/  Rehabilitation | 08/06/2013 | Brisbane | Australia | 1/  Unknown | Bartley et al. 2016 |
|  | LP14 | Environmental | NA | East Wing/  Obstretic | 10/06/2013 | Brisbane | Australia | 1/  Unknown | Bartley et al. 2016 |
|  | LP15 | Environmental | NA | Hyperbaric Unit | 12/06/2013 | Brisbane | Australia | 1/  Unknown | Bartley et al. 2016 |
|  | LP16 | Environmental | NA | Hemato-Oncology Day Facility | 18/06/2013 | Brisbane | Australia | 1/  Unknown | Bartley et al. 2016 |
|  | LP17 | Environmental | NA | Main block/  Medical centre 1 | 21/06/2013 | Brisbane | Australia | 1/  Unknown | Bartley et al. 2016 |
|  | LP18 | Environmental | NA | Main block/  Medical centre 1 | 21/06/2013 | Brisbane | Australia | 1/  Unknown | Bartley et al. 2016 |
|  | LP19 | Environmental | NA | Main block/  Internal medicine | 21/06/2013 | Brisbane | Australia | 1/  Unknown | Bartley et al. 2016 |
|  | LP20 | Environmental | NA | Main block/  Hematology | 21/06/2013 | Brisbane | Australia | 1/  Unknown | Bartley et al. 2016 |
|  | LP21 | Environmental | NA | Main block/  Hematology HDU | 21/06/2013 | Brisbane | Australia | 1/  Unknown | Bartley et al. 2016 |
|  | LP23 | Environmental | NA | Main block/  Cardiac  Catheter Suite | 21/06/2013 | Brisbane | Australia | 1/  Unknown | Bartley et al. 2016 |
|  | LP24 | Environmental | NA | Main block/  Echocardiogr-aphy Laboratory | 21/06/2013 | Brisbane | Australia | 1/  Unknown | Bartley et al. 2016 |
|  | LP25 | Environmental | NA | Main block/  Pediatric | 21/06/2013 | Brisbane | Australia | 1/  Unknown | Bartley et al. 2016 |
|  | LP26 | Environmental | NA | Main block/  Pediatric | 21/06/2013 | Brisbane | Australia | 1/  Unknown | Bartley et al. 2016 |
|  | LP27 | Environmental | NA | Main block/  Pediatric | 21/06/2013 | Brisbane | Australia | 1/  Unknown | Bartley et al. 2016 |
|  | LP28 | Environmental | NA | Main block/  Pediatric | 21/06/2013 | Brisbane | Australia | 1/  Unknown | Bartley et al. 2016 |
|  | LP29 | Environmental | NA | Main block/  Rehabilitation | 21/06/2013 | Brisbane | Australia | 1/  Unknown | Bartley et al. 2016 |
|  | LP30 | Environmental | NA | Main block/  Rehabilitation | 21/06/2013 | Brisbane | Australia | 1/  Unknown | Bartley et al. 2016 |
|  | LP31 | Environmental | NA | Main block/  Rehabilitation | 21/06/2013 | Brisbane | Australia | 1/  Unknown | Bartley et al. 2016 |
|  | LP32 | Environmental | NA | Main block/  Dialysis | 21/06/2013 | Brisbane | Australia | 1/  Unknown | Bartley et al. 2016 |
|  | LP33 | Environmental | NA | Main block/  Radiology | 25/06/2013 | Brisbane | Australia | 1/  Unknown | Bartley et al. 2016 |
|  | LP34 | Environmental | NA | Main block/  Rehabilitation | 28/06/2013 | Brisbane | Australia | 1/  Unknown | Bartley et al. 2016 |
|  | LP35 | Environmental | NA | Main block/  Rehabilitation | 28/06/2013 | Brisbane | Australia | 1/  Unknown | Bartley et al. 2016 |
|  | LP36 | Environmental | NA | Main block/  Rehabilitation | 28/06/2013 | Brisbane | Australia | 1/  Unknown | Bartley et al. 2016 |
|  | LP37 | Environmental | NA | Main block/  Rehabilitation | 05/07/2013 | Brisbane | Australia | 1/  Unknown | Bartley et al. 2016 |
|  | LP38 | Environmental | NA | Main block/  Rehabilitation | 05/07/2013 | Brisbane | Australia | 1/  Unknown | Bartley et al. 2016 |
|  | LP39 | Environmental | NA | Main block/  Rehabilitation | 05/07/2013 | Brisbane | Australia | 1/  Unknown | Bartley et al. 2016 |
|  | LP40 | Environmental | NA | East Wing/  Obstretic | 10/06/2013 | Brisbane | Australia | 1/  Unknown | Bartley et al. 2016 |
|  | LP41 | Environmental | NA | East Wing/  Breast and Endocrine Surgery | 10/06/2013 | Brisbane | Australia | 1/  Unknown | Bartley et al. 2016 |
|  | LP44 (case 8) | Clinical | Hospital B | Main Block/  Hematology HDU | 14/10/2011 | Brisbane | Australia | 1/  Unknown | Bartley et al. 2016 |
|  | LP45 (case 9) | Clinical | Hospital B only | East Wing/  Cardiac | 27/05/2013 | Brisbane | Australia | 1/  Unknown | Bartley et al. 2016 |
|  | LP46 (case 9) | Clinical (from same patient as LP45) |  |  | 31/05/2013 | Brisbane | Australia | 1/  Unknown | Bartley et al. 2016 |
|  | LP47 (case 10) | Clinical | Hospital B only | Main block/  Hematology HDU | 07/06/2013 | Brisbane | Australia | 1/  Unknown | Bartley et al. 2016 |
|  | LP48 (case 10) | Clinical (from same patient as LP47) |  |  | 17/06/2013 | Brisbane | Australia | 1/  Unknown | Bartley et al. 2016 |
| C | Paris (case 11) | Clinical | Hospital C (7 days) & another hospital near to Paris (4 days) | Intensive Care Unit (cardiac surgery) (A) and cardiac surgery unit (B) (Hospital C) | 04/04/2002 | Paris | France | 1/Phil. | Cazalet et al. 2004 |
|  | HL 0051 1015 (case 12) | Clinical | Hospital C only | Nephrology (C) | 12/12/2000 | Paris | France | 1/Phil. | ERR1399510 |
|  | HL 0051 4008 (case 13) | Clinical | Hospital C (~17 days) | Intensive Care Unit (cardiac surgery) (A) | 18/12/2000 | Paris | France | 1/Oxford/OLDA | ERR1399509 |
|  | HL 0101 3003 (case 14) | Clinical | Hospital C (~12 days) | Intensive Care Unit (cardiac surgery) (A) | 27/12/2000 | Paris | France | 1/Phil. | ERR1399564 |
|  | HL 0102 3034 (case 15) | Clinical | Hospital C (~4 days), home | Intensive Care Unit (cardiac surgery) (A) | 21/12/2000 | Paris | France | 1/Phil. | ERR1399514 |
|  | HL 0102 3035 (case 16) | Clinical | Hospital C (~4 days), home | Intensive Care Unit (cardiac surgery) (A) | 24/12/2000 | Paris | France | 1/Phil. | ERR1399517 |
|  | LG 0713 5006 (case 17) | Clinical | Hospital C only | Nephrology (C) | 22/03/2007 | Paris | France | 1/Phil. | ERR1399504 |
|  | HL 0131 3038 | Environmental (shower room 4622) | NA | Oto-Rhino-Laryngology (D) | 01/08/2001 | Paris | France | 1/  Unknown | ERR1399500 |
|  | HL 0131 3039 | Environmental (tap room 4622) | NA | Oto-Rhino-Laryngology (D) | 01/08/2001 | Paris | France | 1/  Unknown | ERR1399502 |
|  | Paris 2001 I n2 | Environmental (room 7411, HWS, shower) | NA | Nephrology (C) | 18/12/2000 | Paris | France | 1/  Unknown | ERR1399552 |
|  | LG 0713 5007 | Environmental (sink 1) | NA | Dialysis room (E) | 25/03/2007 | Paris | France | 1/Oxford/OLDA | ERR1399563 |
|  | LG 0713 5008 | Environmental (sink 3) | NA | Dialysis room (E) | 26/03/2007 | Paris | France | 1/Oxford/OLDA | ERR1399521 |
| D | LG 0918 2002 (case 18) | Clinical | Hospital D (~4 days), home | Internal medicine unit, room 618 (A) | 08/04/2009 | Near Marseille | France | 1/Phil. | ERR1399543 |
|  | LG 1416 4007 (case 19) | Clinical | Hospital D (~3 days), home (~3 days) | Internal medicine unit, room 610 (B) | 10/04/2014 | Near Marseille | France | 1/Phil. | ERR1399515 |
|  | LG 1416 4008 (case 20) | Clinical | Hospital D (~5 days) | Internal medicine unit, room 610 (B) | 07/04/2014 | Near Marseille | France | 1/Phil. | ERR1399571 |
|  | LG 1427 4009 | Environmental | NA | Internal medicine unit, room 610 (B) | 17/06/2014 | Near Marseille | France | 1/Phil. | ERR1399497 |
|  | LG 1427 4010 | Environmental | NA | Internal medicine unit, room 610 (B) | 17/06/2014 | Near Marseille | France | 1/Phil. | ERR1399507 |
|  | LG 0918 2005 | Environmental | NA | Internal medicine unit, room 618 (A) | 16/04/2009 | Near Marseille | France | 1/Phil. | ERR1399529 |
| E | H103120165 (case 21) | Clinical | Hospital E (at least 10 days) | Unknown | 08/06/2010 | London | UK | 1/Phil. | ERR1399524 |
|  | H124240908 (case 22) | Clinical | Hospital E (less than 10 days) | Unknown | 22/10/2012 | London | UK | 1/Phil. | ERR1441930 |
|  | H103340763 | Environmental (sink) | NA | Gastroentero-logy and liver specialist ward (A) | 10/08/2010 | London | UK | 1/Phil. | ERR1399528 |
|  | H124600775 | Environmental | NA | Unknown | 02/11/2012 | London | UK | 1/Phil. | ERR1441929 |
| F | H115180236 (case A) | Clinical | Hospital F (3 days) | Unknown | 26/12/2011 | London | UK | 1/All./  France | ERR1441936 |
| G | H101460286 (case 23) | Clinical | Hospital G (less than 10 days) | Unknown | 02/04/2010 | Cambridge-shire | UK | 1/Oxford/  OLDA | ERR1399527 |
|  | H101740836 | Environmental | NA | Unknown | 20/04/2010 | Cambridge-shire | UK | 1/Oxford/  OLDA | ERR1399532 |
| H | H092520167 (case 24) | Clinical | Hospital H (at least 10 days) | Unknown | 19/06/2009 | London | UK | 1/Oxford/  OLDA | ERR1441933 |
|  | H092620872 (pick 1: H09262087224) | Environmental | NA | Unknown | 19/06/2009 | London | UK | 1/Oxford/  OLDA | ERR1441934 |
| I | H134660746 | Environmental | NA | Unknown | Approx. 14/11/2013 | Essex | UK | 1/Oxford/  OLDA | ERR1441927 |
| J | H102860194 | Clinical | Hospital J (less than 10 days) | Unknown | 20/07/2010 | Near London | UK | 1/Oxford/  OLDA | ERR1441931 |
| K | H074360702 | Environmental | NA | Unknown | 01/10/2007 | Kent | UK | 152/  Oxford/  OLDA | Underwood et al. 2013 |
| L | EUL 55 (case 25) | Clinical | Hospital L | Unknown | 01/04/1994 | Cáceres province | Spain | 1/Oxford/OLDA | David et al. 2016 |
|  | EUL 58 | Environmental | NA | Unknown | 01/01/1994 | Cáceres province | Spain | 1/Oxford/OLDA | David et al. 2016 |
| M | EUL 93 (case 26) | Clinical | Hospital M only | Unknown | 19/10/1992 | Copenhagen | Denmark | 1/Oxford/OLDA | David et al. 2016 |
|  | EUL 94 (case 27) | Clinical | Hospital M only | Unknown | 08/12/1992 | Copenhagen | Denmark | 1/Oxford/OLDA | David et al. 2016 |
|  | EUL 95 | Environmental | NA | Unknown | 21/01/1993 | Copenhagen | Denmark | 1/Oxford/OLDA | David et al. 2016 |
| N | LG 1019 1002 (case 28) | Clinical | Hospital N only | Unknown | 28/04/2010 | Near Marseille | France | 1/Phil. | David et al. 2016 |
|  | LG 1020 3012 | Environmental | NA | Unknown | 27/04/2010 | Near Marseille | France | 1/Phil. | David et al. 2016 |
| O | HL 0311 1005 | Environmental | NA | Room 1010 | 07/03/2003 | Nice | France | 1/Oxford/ OLDA | David et al. 2016 |
| P | EUL 82 | Clinical | Hospital P and home | Unknown | 29/08/1994 | Near Copenhagen | Denmark | 1/Oxford/OLDA | David et al. 2016 |
|  | EUL 85 | Clinical | Hospital P | Unknown | 01/05/1995 | Near Copenhagen | Denmark | 1/Oxford/OLDA | David et al. 2016 |
| Q | EUL 88 | Clinical | Hospital Q only | Unknown | 11/10/1995 | Near  Copenhagen | Denmark | 1/Oxford/OLDA | David et al. 2016 |
| R | LG 1139 1124 | Environmental | NA | Unknown | 14/09/2011 | Near Lyon | France | 1/Oxford/OLDA | David et al. 2016 |
| S/Bundaberg Hospital | LP43 | Clinical | Hospital S | Unknown | 01/03/2001 | Bundaberg | Australia | 1/  Unknown | Bartley et al. 2016 |
| T | L00-549 | Clinical | Hospital T | Unknown | 2000 | Dresden | Germany | 1/  Unknown | David et al. 2016 |
| U | EUL 157 | Environmental | NA | Unknown | 01/07/2004 | Blackpool | UK | 8/  Heysham | David et al. 2016 |
| V | HL 0230 4015 | Clinical | Hospital V (at least 10 days) | Unknown | 23/07/2002 | Near Paris | France | 1/Phil. | David et al. 2016 |
| W | HL 0416 3014 | Clinical | Hospital W (12 days) | Unknown | 25/03/2004 | Brittany | France | 1/Oxford/ OLDA | David et al. 2016 |
| X | LG 1101 1012 | Environmental | NA | Unknown | 09/12/2010 | Haute-Marne region | France | 1/Phil. | David et al. 2016 |
| Y | EUL 16 | Clinical | Hospital Y | Unknown | 06/06/1984 | Glasgow | UK | 5/  Benidorm | David et al. 2016 |
| Z | ID_6885 | Environmental | NA | Unknown | 29/04/2011 | Unknown | Spain | 1/  Unknown | Sanchez-Buso et al. 2014 |
| α | NIIB80 | Clinical | Hospital α | Unknown | 1981 | Nagasaki | Japan | 1/  Unknown | David et al. 2016 |
| ***Isolates from or associated with community sources (i.e. with no links to hospitals) (n=47)*** | | | | | | | | | |
|  | EUL 84 | Clinical |  |  | 03/04/1995 | Unknown | Denmark | 1/Oxford/OLDA | David et al. 2016 |
|  | HL 0036 4001 | Clinical |  |  | 22/08/2000 | Paris | France | 1/Phil. | David et al. 2016 |
|  | HL 0337 3012 | Environmental |  |  | 09/09/2003 | Poitiers | France | 1/  Unknown | David et al. 2016 |
|  | LG 0725 3019 | Environmental |  |  | 04/06/2007 | Poitiers | France | 1/Phil. | ERR1399568 |
|  | LG 0725 3022 | Environmental |  |  | 04/06/2007 | Poitiers | France | 1/  Unknown | ERR1399531 |
|  | LG 1014 3009 | Clinical |  |  | 30/03/2010 | Unknown | France | 1/Oxford/OLDA | David et al. 2016 |
|  | LG 0940 4015 | Clinical |  |  | 24/09/2009 | Lyon | France | 1/Phil. | David et al. 2016 |
|  | Lp-032 | Environmental |  |  | Unknown | Unknown | Israel | 1/  Unknown | Moran-Gilad et al. 2015 |
|  | Lp-119 | Environmental |  |  | 23/04/2013 | Unknown | Israel | 1/  Unknown | Moran-Gilad et al. 2015 |
|  | Lp-120 | Environmental |  |  | 23/04/2013 | Unknown | Israel | 1/  Unknown | Moran-Gilad et al. 2015 |
|  | Lp-121 | Environmental |  |  | 23/04/2013 | Unknown | Israel | 1/  Unknown | Moran-Gilad et al. 2015 |
|  | Lp-122 | Environmental |  |  | 23/04/2013 | Unknown | Israel | 1/  Unknown | Moran-Gilad et al. 2015 |
|  | Lp-2002694p8 | Environmental |  |  | Unknown | Unknown | Israel | 1/Oxford/OLDA | Moran-Gilad et al. 2015 |
|  | Lp-282-1 | Environmental |  |  | 01/08/2013 | Unknown | Israel | 1/  Unknown | Moran-Gilad et al. 2015 |
|  | Lp-283 | Environmental |  |  | 01/08/2013 | Unknown | Israel | 1/  Unknown | Moran-Gilad et al. 2015 |
|  | Lp-284 | Environmental |  |  | 01/08/2013 | Unknown | Israel | 1/  Unknown | Moran-Gilad et al. 2015 |
|  | Lp-285 | Environmental |  |  | 01/08/2013 | Unknown | Israel | 1/  Unknown | Moran-Gilad et al. 2015 |
|  | Lp-286-1 | Environmental |  |  | 01/08/2013 | Unknown | Israel | 1/  Unknown | Moran-Gilad et al. 2015 |
|  | Lp-56207 | Clinical |  |  | Unknown | Unknown | Israel | 1/Oxford/ OLDA | Moran-Gilad et al. 2015 |
|  | EUL 53 | Clinical |  |  | 01/05/1995 | Unknown | Spain | 1/Oxford/OLDA | David et al. 2016 |
|  | ID_1688 | Environmental |  |  | 23/06/2004 | Unknown | Spain | 1/  Unknown | Sanchez-Buso et al. 2014 |
|  | ID_1690 | Environmental |  |  | 23/06/2004 | Unknown | Spain | 1/  Unknown | Sanchez-Buso et al. 2014 |
|  | ID_1828 | Environmental |  |  | 20/09/2004 | Unknown | Spain | 1/  Unknown | Sanchez-Buso et al. 2014 |
|  | ID_2041 | Environmental |  |  | 15/06/2005 | Unknown | Spain | 1/Oxford/OLDA | Sanchez-Buso et al. 2014 |
|  | ID_2947 | Environmental |  |  | 13/06/2000 | Unknown | Spain | 1/Oxford/OLDA | Sanchez-Buso et al. 2014 |
|  | ID_2948 | Environmental |  |  | 13/06/2000 | Unknown | Spain | 1/Oxford/OLDA | Sanchez-Buso et al. 2014 |
|  | ID_598 | Environmental |  |  | 07/02/2002 | Unknown | Spain | 1/Pontiac/Knoxville | Sanchez-Buso et al. 2014 |
|  | ID_747970 | Environmental |  |  | 21/08/2009 | Unknown | Spain | 1/  Unknown | Sanchez-Buso et al. 2014 |
|  | ID_891 | Environmental |  |  | 03/09/2002 | Unknown | Spain | 1/Oxford/OLDA | Sanchez-Buso et al. 2014 |
|  | EUL 104 | Clinical |  |  | 01/01/1992 | Unknown | Sweden | 1/Oxford/OLDA | David et al. 2016 |
|  | EUL 108 | Clinical |  |  | 01/01/1992 | Unknown | Sweden | 1/All./  France | David et al. 2016 |
|  | EUL 1 | Clinical |  |  | 01/02/1998 | Ticino | Switzer-land | 1/Phil. | David et al. 2016 |
|  | EUL 3 | Clinical |  |  | 01/10/1989 | Ticino | Switzer-land | 1/Phil. | David et al. 2016 |
|  | EUL 9 | Environmental |  |  | 01/10/1989 | S. Gallen | Switzer-land | 1/Phil. | David et al. 2016 |
|  | EUL 10 | Environmental |  |  | 01/10/1989 | S. Gallen | Switzer-land | 1/Phil. | David et al. 2016 |
|  | H034800423 | Environmental |  |  | 01/11/2003 | Hereford | UK | 1/Oxford/OLDA | Reuter et al. 2013 |
|  | H072740379 | Environmental (domestic header tank) |  |  | 28/06/2007 | Woking-ham, Berkshire | UK | 1/Phil. | ERR1399548 |
|  | H084800579 | Clinical |  |  | 27/11/2008 | East of England | UK | 1/Oxford/OLDA | ERR1441923 |
|  | H085060063 | Environmental (from home of patient from which above isolate, H084800579, was obtained) |  |  | About 11/12/2008 | Chelmsford, Essex | UK | 1/Oxford/OLDA | ERR1441924 |
|  | H091640624 (case B) | Clinical |  |  | 20/04/2009 | Chelmsford, Essex | UK | 1/Oxford/OLDA | ERR1441928 |
|  | H091720529 | Environmental (from home of patient from which above isolate, H091640624, was obtained) |  |  | 17/04/2009 | East of England | UK | 1/Oxford/OLDA | ERR1441926 |
|  | H100200319 | Environmental (home of case 3) |  |  | 30/12/2009 | Chadwell, Essex | UK | 1/Oxford/OLDA | ERR1399534 |
|  | H100200320 | Environmental (home of case 3) |  |  | 30/12/2009 | Chadwell, Essex | UK | 1/Oxford/OLDA | ERR1399538 |
|  | H100200321 | Environmental (home of case 3) |  |  | 30/12/2009 | Chadwell, Essex | UK | 1/NA (mAb all negative) | ERR1399541 |
|  | H115260949 | Environmental (home of case A who also spent part of their incubation period in Hospital F) |  |  | 26/12/2011 | London | UK | 1/Phil. | ERR1441935 |
|  | H152640286 (case C) | Clinical |  |  | 22/06/2015 | East of England | UK | 1/Oxford/OLDA | ERR1441925 |
|  | H152780272 | Environmental (from home of patient from which above isolate, H152780272, was obtained) |  |  | 01/07/2015 | East of England | UK | 1/Oxford/OLDA | ERR1441932 |
| ***Isolates from a cruise ship (n=3)*** | | | | | | | | | |
|  | H073300077 | Environmental |  |  | Approx. 8/8/2007 | NA | NA | 1/Oxford/OLDA | ERR1399519 |
|  | H073300079 | Environmental |  |  | Approx. 8/8/2007 | NA | NA | 1/Oxford/OLDA | ERR1399496 |
|  | H073360657 | Environmental |  |  | Approx. 5/8/2007 | NA | NA | 1/Oxford/OLDA | ERR1399557 |
| ***Isolates with an unknown sampling context (n=38)*** | | | | | | | | | |
|  | L 3386/03 | Environmental |  |  | 2003 | Unknown | Austria | 1/  Unknown | David et al. 2016 |
|  | L 3415/03 | Environmental |  |  | 2003 | Unknown | Austria | 1/  Unknown | David et al. 2016 |
|  | LT 40/04 | Clinical |  |  | 2004 | Unknown | Austria | 1/  Unknown | David et al. 2016 |
|  | Wien 47-14 | Environmental |  |  | 1996 | Unknown | Austria | 1/  Unknown | David et al. 2016 |
|  | EUL 90 | Clinical |  |  | Unknown | Unknown | Denmark | 1/Oxford/ OLDA | David et al. 2016 |
|  | E21203 | Clinical |  |  | 2004 | Unknown | France | 1/  Unknown | David et al. 2016 |
|  | HL 0701 3004 | Environmental |  |  | 03/01/2007 | Rueil Malmaison | France | 1/Oxford/OLDA | David et al. 2016 |
|  | LG 0919 2006 | Clinical |  |  | 23/04/2009 | Saint Nazaire | France | 1/Phil. | David et al. 2016 |
|  | LG 1105 4025 | Environmental |  |  | 19/01/2011 | Unknown | France | 1/Phil. | David et al. 2016 |
|  | EUL 110 | Clinical |  |  | 01/01/1993 | Luebeck | Germany | 10/Oxford/OLDA | David et al. 2016 |
|  | EUL 113 | Environmental |  |  | 27/02/1995 | Hannover | Germany | 7/Oxford/OLDA | David et al. 2016 |
|  | EUL 114 | Environmental |  |  | 27/02/1995 | Hannover | Germany | 7/Oxford/OLDA | David et al. 2016 |
|  | EUL 117 | Clinical |  |  | 01/06/2005 | Unknown | Germany | 6/  Benidorm | David et al. 2016 |
|  | EUL 119 | Clinical |  |  | 01/06/2005 | Unknown | Germany | 1/Oxford/OLDA | David et al. 2016 |
|  | EUL 60 | Clinical |  |  | 01/01/1992 | Unknown | Greece | 1/Phil. | David et al. 2016 |
|  | EUL 62 | Environmental |  |  | 01/01/1989 | Unknown | Greece | 1/Oxford/OLDA | David et al. 2016 |
|  | EUL 67 | Clinical |  |  | 01/01/1995 | Unknown | Greece | 1/Oxford/OLDA | David et al. 2016 |
|  | EUL 37 | Clinical |  |  | 01/01/1999 | Unknown | Italy | 1/Phil. | David et al. 2016 |
|  | EUL 42 | Clinical |  |  | 01/01/1999 | Unknown | Italy | 1/Phil. | David et al. 2016 |
|  | EUL 43 | Clinical |  |  | 01/01/1999 | Unknown | Italy | 1/Phil. | David et al. 2016 |
|  | EUL 44 | Environmental |  |  | 01/01/1999 | Unknown | Italy | 1/Phil. | David et al. 2016 |
|  | EUL 45 | Clinical |  |  | 01/01/1999 | Unknown | Italy | 72/Phil. | David et al. 2016 |
|  | EUL 46 | Environmental |  |  | 01/01/1999 | Unknown | Italy | 1/Oxford/OLDA | David et al. 2016 |
|  | NIIB223 | Environmental |  |  | 1986 | Unknown | Japan | 1/  Unknown | David et al. 2016 |
|  | NIIB225 | Environmental |  |  | 1986 | Unknown | Japan | 1/  Unknown | David et al. 2016 |
|  | LG 1118 1044 | Environmental |  |  | 11/07/2009 | Unknown | Morocco | 1/Oxford/OLDA | David et al. 2016 |
|  | ATCC 35289 | Environmental |  |  | 1988 | Unknown | Nether-lands | 390/NA (sg9) | David et al. 2016 |
|  | EUL 109 | Environmental |  |  | 01/01/1992 | Unknown | Sweden | 1/Oxford/OLDA | David et al. 2016 |
|  | LP21  (LP21_  Sweden) | Clinical |  |  | 1996-1999 | Unknown | Sweden | 1/  Unknown | David et al. 2016 |
|  | LP22  (LP22_  Sweden) | Clinical |  |  | 1996-1999 | Unknown | Sweden | 1/  Unknown | David et al. 2016 |
|  | LP23  (LP23_  Sweden) | Clinical |  |  | 1996-2000 | Unknown | Sweden | 1/  Unknown | David et al. 2016 |
|  | EUL 13 | Clinical |  |  | 01/01/1994 | Unknown | UK | 1/  Benidorm | David et al. 2016 |
|  | EUL 14 | Clinical |  |  | 06/06/1984 | Glasgow | UK | 5/  Benidorm | David et al. 2016 |
|  | EUL 17 | Clinical |  |  | 01/01/1993 | Ayrshire | UK | 7/Phil. | David et al. 2016 |
|  | EUL 21 | Environmental |  |  | 01/01/1999 | Glasgow | UK | 1/Phil. | David et al. 2016 |
|  | H103620682 | Environmental |  |  | 20/07/2010 | Near London | UK | 1/Oxford/  OLDA | ERR1441922 |
|  | 2735 | Environmental |  |  | 2002 | Unknown | USA | 1/  Unknown | David et al. 2016 |
|  | OLDA1 (NCTC12008) | Clinical |  |  | 1947 | Washington | USA | 1/Oxford/OLDA | David et al. 2016 |

**Table S2. Recombined regions in the ST1 lineage with respect to the coordinates in the Paris genome.**

| **Region start** | **Region end** | **Length of recombined region (bp)** | **Affected taxa** |
| --- | --- | --- | --- |
| 888224 | 955432 | 67209 | EUL 117, L3415-03, L3386-03 |
| 964978 | 990570 | 25593 | EUL 117, L3415-03, L3386-03 |
| 1994336 | 2003075 | 8740 | EUL 117, L3415-03, L3386-03 |
| 471846 | 488208 | 16363 | EUL 117, L3415-03, L3386-03 |
| 500118 | 510563 | 10446 | EUL 117, L3415-03, L3386-03 |
| 1994399 | 1998121 | 3723 | H034800423 |
| 7231 | 40824 | 33594 | EUL 157 |
| 854668 | 944969 | 90302 | EUL157 |
| 1051130 | 1085521 | 34392 | EUL 157 |
| 1991641 | 2001091 | 9451 | EUL 157 |
| 1110682 | 1138668 | 27987 | EUL 157 |
| 2958721 | 2970009 | 11289 | EUL 157 |
| 976443 | 990189 | 13747 | EUL 157 |
| 1793798 | 1815096 | 21299 | EUL 157 |
| 1173689 | 1174215 | 527 | EUL 157 |
| 946171 | 953507 | 7337 | EUL 157 |
| 1994399 | 1998121 | 3723 | Lp-032 |
| 917120 | 932822 | 15703 | L00-549 |
| 425877 | 432715 | 6839 | L00-549 |
| 899423 | 931305 | 31883 | EUL 114, EUL 113 |
| 190429 | 190457 | 29 | EUL 114, EUL 113 |
| 696167 | 755335 | 59169 | EUL 110 |
| 775012 | 777299 | 2288 | EUL 110 |
| 948967 | 952146 | 3180 | EUL 110 |
| 3376219 | 3398926 | 22708 | HL 0701 3004 |
| 1454818 | 1470730 | 15913 | HL 0701 3004 |
| 3360975 | 3365498 | 4524 | HL 0701 3004 |
| 914955 | 967396 | 52442 | ATCC 35289 |
| 720073 | 720104 | 32 | EUL 46 |
| 1173689 | 1174171 | 483 | ID_6885 |
| 1765612 | 1766927 | 1316 | ID_6885 |
| 719005 | 720312 | 1308 | ID_6885, ATCC 35289, EUL 46 |
| 1628854 | 1634840 | 5987 | LG 1014 3009 |
| 1154905 | 1159167 | 4263 | LG 1014 3009 |
| 1994399 | 1998121 | 3723 | LG 1014 3009 |
| 2258179 | 2309479 | 51301 | EUL 110, HL 0701 3004, ID_6885, ATCC 35289, EUL 46 |
| 1988947 | 2036482 | 47536 | EUL 110, HL 0701 3004, ID_6885, ATCC 35289, EUL 46 |
| 613529 | 624131 | 10603 | EUL 110, HL 0701 3004, ID_6885, ATCC 35289, EUL 46 |
| 713674 | 720333 | 6660 | EUL 110, HL 0701 3004, ID_6885, ATCC 35289, EUL 46 |
| 1782638 | 1811993 | 29356 | EUL 110, HL 0701 3004, ID_6885, ATCC 35289, EUL 46 |
| 917023 | 933108 | 16086 | EUL 110, HL 0701 3004, ID_6885, ATCC 35289, EUL 46 |
| 568300 | 576793 | 8494 | EUL 110, HL 0701 3004, ID_6885, ATCC 35289, EUL 46 |
| 2685258 | 2688678 | 3421 | EUL 110, HL 0701 3004, ID_6885, ATCC 35289, EUL 46 |
| 1304731 | 1326450 | 21720 | EUL 110, HL 0701 3004, ID_6885, ATCC 35289, EUL 46 |
| 2067801 | 2071190 | 3390 | EUL 110, HL 0701 3004, ID_6885, ATCC 35289, EUL 46 |
| 789607 | 797972 | 8366 | EUL 110, HL 0701 3004, ID_6885, ATCC 35289, EUL 46 |
| 1135667 | 1142949 | 7283 | EUL 110, HL 0701 3004, ID_6885, ATCC 35289, EUL 46 |
| 1838009 | 1839319 | 1311 | EUL 110, HL 0701 3004, ID_6885, ATCC 35289, EUL 46 |
| 1068888 | 1075051 | 6164 | EUL 110, HL 0701 3004, ID_6885, ATCC 35289, EUL 46 |
| 1250656 | 1254075 | 3420 | EUL 110, HL 0701 3004, ID_6885, ATCC 35289, EUL 46 |
| 2703422 | 2708556 | 5135 | LP37, LP31 |
| 2684281 | 2688323 | 4043 | LP41 |
| 2702109 | 2711198 | 9090 | LP40 |
| 2700766 | 2709758 | 8993 | LP40, LP41, LP45, LP46 |
| 2683386 | 2689185 | 5800 | LP40, LP41, LP45, LP46 |
| 2697841 | 2698121 | 281 | LP20 |
| 2684185 | 2689467 | 5283 | LP37, LP31, LP30, LP40, LP41, LP45, LP46, LP20 |
| 2703398 | 2708855 | 5458 | LP37, LP31, LP30, LP40, LP41, LP45, LP46, LP20 |
| 2661767 | 2691391 | 29625 | LP15 |
| 2707809 | 2707881 | 73 | LP15 |
| 2678351 | 2682061 | 3711 | LP23 |
| 2677952 | 2685270 | 7319 | LP08 |
| 2679495 | 2686677 | 7183 | LP33, LP23 |
| 2678042 | 2687942 | 9901 | LP44, LP06, LP33, LP23, LP48, LP47, LP07, LP08 |
| 2697841 | 2699326 | 1486 | LP44, LP06, LP33, LP23, LP48, LP47, LP07, LP08 |
| 2683386 | 2692896 | 9511 | LP15, LP14, LP37, LP31, LP30, LP40, LP41, LP45, LP46, LP20, LP02, LP04, LP03, LP01 |
| 2710695 | 2711310 | 616 | LP15, LP14, LP37, LP31, LP30, LP40, LP41, LP45, LP46, LP20, LP02, LP04, LP03, LP01 |
| 1561107 | 1561126 | 20 | LP15, LP14, LP37, LP31, LP30, LP40, LP41, LP45, LP46, LP20, LP02, LP04, LP03, LP01 |
| 1587187 | 1593216 | 6030 | LP15, LP14, LP37, LP31, LP30, LP40, LP41, LP45, LP46, LP20, LP02, LP04, LP03, LP01 |
| 1555478 | 1597628 | 42151 | LP15, LP14, LP37, LP31, LP30, LP40, LP41, LP45, LP46, LP20, LP02, LP04, LP03, LP01, LP05, LP24, LP10, LP27, LP26, LP28, LP19, LP16, LP18, LP25, LP39, LP38, LP11, LP34, LP36, LP29, LP35, LP12, LP17, LP09, LP32, LP21, LP44, LP06, LP33, LP23, LP48, LP47, LP07, LP08 |
| 1057185 | 1064266 | 7082 | LP15, LP14, LP37, LP31, LP30, LP40, LP41, LP45, LP46, LP20, LP02, LP04, LP03, LP01, LP05, LP24, LP10, LP27, LP26, LP28, LP19, LP16, LP18, LP25, LP39, LP38, LP11, LP34, LP36, LP29, LP35, LP12, LP17, LP09, LP32, LP21, LP44, LP06, LP33, LP23, LP48, LP47, LP07, LP08 |
| 658706 | 663960 | 5255 | LP15, LP14, LP37, LP31, LP30, LP40, LP41, LP45, LP46, LP20, LP02, LP04, LP03, LP01, LP05, LP24, LP10, LP27, LP26, LP28, LP19, LP16, LP18, LP25, LP39, LP38, LP11, LP34, LP36, LP29, LP35, LP12, LP17, LP09, LP32, LP21, LP44, LP06, LP33, LP23, LP48, LP47, LP07, LP08 |
| 536255 | 537152 | 898 | LP15, LP14, LP37, LP31, LP30, LP40, LP41, LP45, LP46, LP20, LP02, LP04, LP03, LP01, LP05, LP24, LP10, LP27, LP26, LP28, LP19, LP16, LP18, LP25, LP39, LP38, LP11, LP34, LP36, LP29, LP35, LP12, LP17, LP09, LP32, LP21, LP44, LP06, LP33, LP23, LP48, LP47, LP07, LP08 |
| 563811 | 563844 | 34 | LP15, LP14, LP37, LP31, LP30, LP40, LP41, LP45, LP46, LP20, LP02, LP04, LP03, LP01, LP05, LP24, LP10, LP27, LP26, LP28, LP19, LP16, LP18, LP25, LP39, LP38, LP11, LP34, LP36, LP29, LP35, LP12, LP17, LP09, LP32, LP21, LP44, LP06, LP33, LP23, LP48, LP47, LP07, LP08 |
| 2657113 | 2746533 | 89421 | ID_2041 |
| 1173689 | 1192603 | 18915 | ID_2041 |
| 1693701 | 1694442 | 742 | ID_2041 |
| 1997471 | 2000302 | 2832 | ID_2041, ID_1688 |
| 1982506 | 2005678 | 23173 | ID_747970 |
| 2960175 | 2969382 | 9208 | ID_747970 |
| 1838063 | 1846349 | 8287 | ID_747970 |
| 1994399 | 1998121 | 3723 | LG 1139 1124, LP15, LP14, LP37, LP31, LP30, LP40, LP41, LP45, LP46, LP20, LP02, LP04, LP03, LP01, LP05, LP24, LP10, LP27, LP26, LP28, LP19, LP16, LP18, LP25, LP39, LP38, LP11, LP34, LP36, LP29, LP35, LP12, LP17, LP09, LP32, LP21, LP44, LP06, LP33, LP23, LP48, LP47, LP07, LP08, ID_2041, ID_1688, ID_747970, Lp-2002694p8, Lp-56207 |
| 1994432 | 2001118 | 6687 | L00-549, LG 1118 1044, EUL 119, EUL 114, EUL 113 |
| 23703 | 41752 | 18050 | L00-549, LG 1118 1044, EUL 119, EUL 114, EUL 113 |
| 54158 | 60859 | 6702 | L00-549, LG 1118 1044, EUL 119, EUL 114, EUL 113 |
| 622300 | 628159 | 5860 | L00-549, LG 1118 1044, EUL 119, EUL 114, EUL 113 |
| 1818963 | 1822135 | 3173 | L00-549, LG 1118 1044, EUL 119, EUL 114, EUL 113 |
| 2504111 | 2504125 | 15 | LG 1014 3009, EUL 110, HL 0701 3004, ID_6885, ATCC 35289, EUL 46, LG 1139 1124, LP15, LP14, LP37, LP31, LP30, LP40, LP41, LP45, LP46, LP20, LP02, LP04, LP03, LP01, LP05, LP24, LP10, LP27, LP26, LP28, LP19, LP16, LP18, LP25, LP39, LP38, LP11, LP34, LP36, LP29, LP35, LP12, LP17, LP09, LP32, LP21, LP44, LP06, LP33, LP23, LP48, LP47, LP07, LP08, ID_2041, ID_1688, ID_747970, Lp-2002694p8, Lp-56207 |
| 838377 | 919389 | 81013 | H074360702 |
| 3049179 | 3101539 | 52361 | H074360702 |
| 23516 | 27518 | 4003 | H074360702 |
| 110845 | 113854 | 3010 | H074360702 |
| 699197 | 699401 | 205 | H074360702 |
| 737148 | 755353 | 18206 | H134660746 |
| 789602 | 806513 | 16912 | H134660746 |
| 1992350 | 2001487 | 9138 | H101460286, H101740836 |
| 137388 | 167272 | 29885 | H074360702, H134660746 |
| 1983181 | 2000749 | 17569 | H074360702, H134660746 |
| 1915110 | 1916841 | 1732 | H074360702, H134660746 |
| 1060915 | 1114436 | 53522 | 2735 |
| 2392732 | 2421895 | 29164 | 2735 |
| 993110 | 1038854 | 45745 | 2735 |
| 298539 | 314989 | 16451 | 2735 |
| 1982357 | 2017240 | 34884 | 2735 |
| 3363756 | 3413651 | 49896 | 2735 |
| 1802571 | 1857618 | 55048 | 2735 |
| 2530045 | 2549526 | 19482 | 2735 |
| 2280942 | 2293075 | 12134 | 2735 |
| 832911 | 837701 | 4791 | 2735 |
| 2220837 | 2224037 | 3201 | 2735 |
| 3457154 | 3457924 | 771 | 2735 |
| 392607 | 414711 | 22105 | H101460286, H101740836, H074360702, H134660746 |
| 351708 | 388971 | 37264 | H101460286, H101740836, H074360702, H134660746 |
| 421687 | 432143 | 10457 | H101460286, H101740836, H074360702, H134660746 |
| 68804 | 69392 | 589 | H101460286, H101740836, H074360702, H134660746 |
| 440235 | 445176 | 4942 | H101460286, H101740836, H074360702, H134660746 |
| 737046 | 755336 | 18291 | H092520167, H092620872 |
| 775012 | 775552 | 541 | H092520167, H092620872 |
| 2717875 | 2723541 | 5667 | H092520167, H092620872 |
| 2693167 | 2694304 | 1138 | H073300079, H073300077, H073360657 |
| 2739452 | 2739488 | 37 | H073300079, H073300077, H073360657 |
| 2669078 | 2669152 | 75 | H092520167, H092620872, H103620682, H102860194 |
| 2714717 | 2724789 | 10073 | Lp-284, Lp-282-1 |
| 207176 | 213304 | 6129 | Lp-284, Lp-282-1 |
| 2691496 | 2700180 | 8685 | Lp-284, Lp-282-1 |
| 193290 | 193449 | 160 | Lp-284, Lp-282-1 |
| 1193155 | 1203231 | 10077 | Lp-284, Lp-282-1 |
| 2670006 | 2670032 | 27 | Lp-284, Lp-282-1 |
| 872498 | 906144 | 33647 | H073300079, H073300077, H073360657, H092520167, H092620872, H103620682, H102860194, Lp-284, Lp-282-1 |
| 1900974 | 1917563 | 16590 | H073300079, H073300077, H073360657, H092520167, H092620872, H103620682, H102860194, Lp-284, Lp-282-1 |
| 1988831 | 2008972 | 20142 | H073300079, H073300077, H073360657, H092520167, H092620872, H103620682, H102860194, Lp-284, Lp-282-1 |
| 1981851 | 2025625 | 43775 | EUL 90, EUL 82, EUL 85, EUL 88, EUL 95, EUL 94, EUL 93, EUL 84 |
| 2692510 | 2693776 | 1267 | EUL 90, EUL 82, EUL 85, EUL 88, EUL 95, EUL 94, EUL 93, EUL 84 |
| 2717817 | 2724132 | 6316 | EUL 90, EUL 82, EUL 85, EUL 88, EUL 95, EUL 94, EUL 93, EUL 84 |
| 182787 | 219994 | 37208 | H073300079, H073300077, H073360657, H092520167, H092620872, H103620682, H102860194, Lp-284, Lp-282-1, EUL 90, EUL 82, EUL 85, EUL 88, EUL 95, EUL 94, EUL 93, EUL 84 |
| 2665989 | 2699050 | 33062 | H073300079, H073300077, H073360657, H092520167, H092620872, H103620682, H102860194, Lp-284, Lp-282-1, EUL 90, EUL 82, EUL 85, EUL 88, EUL 95, EUL 94, EUL 93, EUL 84 |
| 1163555 | 1203333 | 39779 | H073300079, H073300077, H073360657, H092520167, H092620872, H103620682, H102860194, Lp-284, Lp-282-1, EUL 90, EUL 82, EUL 85, EUL 88, EUL 95, EUL 94, EUL 93, EUL 84 |
| 2709950 | 2724645 | 14696 | H073300079, H073300077, H073360657, H092520167, H092620872, H103620682, H102860194, Lp-284, Lp-282-1, EUL 90, EUL 82, EUL 85, EUL 88, EUL 95, EUL 94, EUL 93, EUL 84 |
| 1986115 | 2009403 | 23289 | H073300079, H073300077, H073360657, H092520167, H092620872, H103620682, H102860194, Lp-284, Lp-282-1, EUL 90, EUL 82, EUL 85, EUL 88, EUL 95, EUL 94, EUL 93, EUL 84 |
| 2700732 | 2700742 | 11 | H073300079, H073300077, H073360657, H092520167, H092620872, H103620682, H102860194, Lp-284, Lp-282-1, EUL 90, EUL 82, EUL 85, EUL 88, EUL 95, EUL 94, EUL 93, EUL 84 |
| 2007139 | 2149829 | 142691 | H085060063 H084800579 |
| 2228527 | 2232495 | 3969 | H085060063 H084800579 |
| 2273096 | 2301712 | 28617 | H085060063 H084800579 |
| 1184767 | 1193405 | 8639 | H085060063 H084800579 |
| 2160967 | 2167895 | 6929 | H085060063 H084800579 |
| 1100245 | 1120684 | 20440 | H085060063 H084800579 |
| 1994529 | 2005198 | 10670 | H085060063 H084800579 |
| 2709947 | 2722846 | 12900 | H085060063 H084800579 |
| 1663229 | 1666442 | 3214 | H085060063 H084800579 |
| 1594005 | 1610291 | 16287 | H085060063 H084800579 |
| 1098710 | 1099112 | 403 | H085060063 H084800579 |
| 68053 | 76221 | 8169 | 2735, H101460286, H101740836, H074360702, H134660746 |
| 1205044 | 1211176 | 6133 | 2735, H101460286, H101740836, H074360702, H134660746 |
| 2677346 | 2724618 | 47273 | H073300079, H073300077, H073360657, H092520167, H092620872, H103620682, H102860194, Lp-284, Lp-282-1, EUL 90, EUL 82, EUL 85, EUL 88, EUL 95, EUL 94, EUL 93, EUL 84, H085060063, H084800579 |
| 1160175 | 1203349 | 43175 | H073300079, H073300077, H073360657, H092520167, H092620872, H103620682, H102860194, Lp-284, Lp-282-1, EUL 90, EUL 82, EUL 85, EUL 88, EUL 95, EUL 94, EUL 93, EUL 84, H085060063, H084800579 |
| 2661627 | 2665974 | 4348 | H073300079, H073300077, H073360657, H092520167, H092620872, H103620682, H102860194, Lp-284, Lp-282-1, EUL 90, EUL 82, EUL 85, EUL 88, EUL 95, EUL 94, EUL 93, EUL 84, H085060063, H084800579 |
| 1995251 | 2009451 | 14201 | H073300079, H073300077, H073360657, H092520167, H092620872, H103620682, H102860194, Lp-284, Lp-282-1, EUL 90, EUL 82, EUL 85, EUL 88, EUL 95, EUL 94, EUL 93, EUL 84, H085060063, H084800579 |
| 1454845 | 1458138 | 3294 | H073300079, H073300077, H073360657, H092520167, H092620872, H103620682, H102860194, Lp-284, Lp-282-1, EUL 90, EUL 82, EUL 85, EUL 88, EUL 95, EUL 94, EUL 93, EUL 84, H085060063, H084800579 |
| 3310617 | 3319330 | 8714 | EUL 21 |
| 2994178 | 3006241 | 12064 | EUL 21 |
| 3162890 | 3194513 | 31624 | EUL 21 |
| 2954911 | 2963935 | 9025 | EUL 21 |
| 3091916 | 3106059 | 14144 | EUL 21 |
| 3228756 | 3249903 | 21148 | EUL 21 |
| 3422165 | 3423998 | 1834 | EUL 21 |
| 2520359 | 2529673 | 9315 | EUL 21 |
| 3149042 | 3155746 | 6705 | EUL 21 |
| 915120 | 955337 | 40218 | EUL 14, EUL 13, EUL 16 |
| 6516 | 34195 | 27680 | EUL 14, EUL 13, EUL 16 |
| 2895018 | 2895040 | 23 | EUL 14, EUL 13, EUL 16 |
| 1998845 | 1998859 | 15 | EUL 14, EUL 13, EUL 16 |
| 404935 | 406960 | 2026 | EUL 14, EUL 13, EUL 16 |
| 136636 | 150768 | 14133 | EUL 14, EUL 13, EUL 16 |
| 607809 | 619627 | 11819 | EUL 14, EUL 13, EUL 16 |
| 642420 | 643590 | 1171 | EUL 14, EUL 13, EUL 16 |
| 190375 | 190387 | 13 | Lp-285 |
| 3061567 | 3069264 | 7698 | Lp-285, Lp-283 |
| 3061410 | 3069294 | 7885 | Lp-286-1 |
| 3061567 | 3069264 | 7698 | Lp-120, Lp-119, Lp-121, Lp-122 |
| 1981331 | 2027320 | 45990 | EUL 53 |
| 2074251 | 2094482 | 20232 | EUL 53 |
| 1921849 | 1928683 | 6835 | EUL 53 |
| 2144221 | 2147242 | 3022 | EUL 53 |
| 1992861 | 2004210 | 11350 | NIIB223, NIIB225, NIIB80 |
| 2959733 | 2968259 | 8527 | NIIB223, NIIB225, NIIB80 |
| 926478 | 932256 | 5779 | NIIB223, NIIB225, NIIB80 |
| 648716 | 650067 | 1352 | NIIB223, NIIB225, NIIB80 |
| 1159937 | 1165511 | 5575 | NIIB223, NIIB225, NIIB80 |
| 190502 | 190530 | 29 | EUL 62 |
| 3061410 | 3069294 | 7885 | NIIB223, NIIB225, NIIB80, OLDA1, EUL 53, ID_1690, EUL 62, EUL 67 |
| 3061567 | 3069264 | 7698 | EUL 58, EUL 55 |
| 1058495 | 1070318 | 11824 | LP43 |
| 1981424 | 2035681 | 54258 | LT40-04 |
| 2294421 | 2307736 | 13316 | LT40-04 |
| 2365579 | 2377087 | 11509 | LT40-04 |
| 1911568 | 1921849 | 10282 | LT40-04 |
| 2884279 | 2905847 | 21569 | LT40-04 |
| 2956905 | 2965649 | 8745 | LT40-04 |
| 942922 | 944078 | 1157 | ID_891, ID_598, ID_2947 |
| 2914288 | 2914308 | 21 | H124240908, H124600775 |
| 2895697 | 2932766 | 37070 | H124240908, H124600775, H103120165, H103340763 |
| 3052827 | 3085034 | 32208 | H124240908, H124600775, H103120165, H103340763 |
| 3785 | 13718 | 9934 | H124240908, H124600775, H103120165, H103340763 |
| 1992350 | 2003075 | 10726 | H124240908, H124600775, H103120165, H103340763 |
| 3115071 | 3117964 | 2894 | H124240908, H124600775, H103120165, H103340763 |
| 191429 | 191461 | 33 | H124240908, H124600775, H103120165, H103340763 |
| 858081 | 933217 | 75137 | EUL 17 |
| 791380 | 807526 | 16147 | EUL 17 |
| 1983181 | 2000749 | 17569 | EUL 17 |
| 2484447 | 2490279 | 5833 | EUL 17 |
| 299130 | 309007 | 9878 | EUL 109, EUL 104, LP22_Sweden, LP23_Sweden, LP21_Sweden, EUL 108 |
| 915641 | 929335 | 13695 | EUL 109, EUL 104, LP22_Sweden, LP23_Sweden, LP21_Sweden, EUL 108 |
| 2422757 | 2427342 | 4586 | EUL 109, EUL 104, LP22_Sweden, LP23_Sweden, LP21_Sweden, EUL 108 |
| 1988617 | 1988653 | 37 | EUL 109, EUL 104, LP22_Sweden, LP23_Sweden, LP21_Sweden, EUL 108 |
| 2100477 | 2103214 | 2738 | EUL 109, EUL 104, LP22_Sweden, LP23_Sweden, LP21_Sweden, EUL 108 |
| 1682116 | 1852658 | 170543 | HL 0416 3014 |
| 1078977 | 1114651 | 35675 | HL 0416 3014 |
| 1041081 | 1069391 | 28311 | HL 0416 3014 |
| 2895553 | 2902596 | 7044 | HL 0416 3014 |
| 2526748 | 2530864 | 4117 | HL 0416 3014 |
| 1653996 | 1658370 | 4375 | HL 0416 3014 |
| 529824 | 535157 | 5334 | HL 0416 3014 |
| 922988 | 929825 | 6838 | HL 0416 3014 |
| 394532 | 396154 | 1623 | HL 0416 3014 |
| 1024494 | 1027312 | 2819 | H072740379 |
| 1682466 | 1685185 | 2720 | H072740379 |
| 1003168 | 1003310 | 143 | H072740379 |
| 917563 | 929173 | 11611 | EUL 109, EUL 104, LP22_Sweden, LP23_Sweden, LP21_Sweden, EUL 108, HL 0416 3014 |
| 396336 | 419975 | 23640 | EUL 42 |
| 1995263 | 2005158 | 9896 | EUL 42 |
| 190429 | 190457 | 29 | EUL 42 |
| 1982318 | 1989642 | 7325 | EUL 42 |
| 393610 | 420389 | 26780 | EUL 42, HL 0230 4015, LG 1020 3012, LG 1019 1002, HL 0311 1005, LG 1101 1012 |
| 1981678 | 2012765 | 31088 | EUL 42, HL 0230 4015, LG 1020 3012, LG 1019 1002, HL 0311 1005, LG 1101 1012 |
| 1981796 | 2013225 | 31430 | LG 0940 4015, EUL 42, HL 0230 4015, LG 1020 3012, LG 1019 1002, HL 0311 1005, LG 1101 1012 |
| 392896 | 420410 | 27515 | LG 0940 4015, EUL 42, HL 0230 4015, LG 1020 3012, LG 1019 1002, HL 0311 1005, LG 1101 1012 |
| 1997192 | 2007119 | 9928 | ID_1828 |
| 3164825 | 3184005 | 19181 | ID_1828 |
| 797766 | 800816 | 3051 | ID_1828 |
| 1997091 | 2040992 | 43902 | ID_1828, E21203 |
| 3164330 | 3184696 | 20367 | ID_1828, E21203 |
| 795565 | 800955 | 5391 | ID_1828, E21203 |
| 855034 | 857001 | 1968 | ID_1828, E21203 |
| 823882 | 833348 | 9467 | ID_1828, E21203 |
| 1981457 | 2018046 | 36590 | H072740379, EUL 109, EUL 104, LP22_Sweden, LP23_Sweden, LP21_Sweden, EUL 108, HL 0416 3014 |
| 843689 | 861966 | 18278 | H072740379, EUL 109, EUL 104, LP22_Sweden, LP23_Sweden, LP21_Sweden, EUL 108, HL 0416 3014 |
| 877964 | 891092 | 13129 | H072740379, EUL 109, EUL 104, LP22_Sweden, LP23_Sweden, LP21_Sweden, EUL 108, HL 0416 3014 |
| 2091402 | 2104212 | 12811 | H072740379, EUL 109, EUL 104, LP22_Sweden, LP23_Sweden, LP21_Sweden, EUL 108, HL 0416 3014 |
| 926718 | 928280 | 1563 | H072740379, EUL 109, EUL 104, LP22_Sweden, LP23_Sweden, LP21_Sweden, EUL 108, HL 0416 3014 |
| 1992558 | 2001487 | 8930 | Wien_47-14, HL 0036 4001, Paris, HL 0101 3003, HL 0102 3034, HL 0102 3035, Paris_2001_I_n2, HL 0051 1015, HL 0131 3039, LG 0713 5006, LG 0713 5007, EUL 3, EUL 10, EUL 9, LG 0940 4015, EUL 42, HL 0230 4015, LG 1020 3012, LG 1019 1002, HL 0311 1005, LG 1101 1012, LG 1105 4025, LG 0919 2006, HL 0337 3012, LG 0725 3019, LG 0725 3022, LG 1427 4010, LG 1427 4009, LG 0918 2002, LG 0918 2005, LG 1416 4007, LG 1416 4008, ID_1828, E21203 |
| 3380081 | 3395276 | 15196 | EUL 44 |
| 2860553 | 2909359 | 48807 | EUL 37 |
| 2966569 | 2988686 | 22118 | EUL 37 |
| 271865 | 291538 | 19674 | EUL 37 |
| 351098 | 365245 | 14148 | EUL 37 |
| 591359 | 610326 | 18968 | EUL 37 |
| 3387578 | 3396011 | 8434 | EUL 37 |
| 2918024 | 2927687 | 9664 | EUL 37 |
| 254646 | 262413 | 7768 | EUL 37 |
| 2939203 | 2945769 | 6567 | EUL 37 |
| 95817 | 105285 | 9469 | EUL 37 |
| 1465283 | 1470416 | 5134 | EUL 37 |
| 787084 | 789218 | 2135 | EUL 37 |
| 28886 | 30371 | 1486 | EUL 37 |
| 1981994 | 2008857 | 26864 | EUL 43 |
| 256074 | 264516 | 8443 | EUL 37, EUL 44, EUL 45 |
| 1992558 | 2015013 | 22456 | EUL 37, EUL 44, EUL 45 |
| 3393726 | 3395209 | 1484 | EUL 37, EUL 44, EUL 45 |
| 1011977 | 1012018 | 42 | H072560534 |
| 1055652 | 1093673 | 38022 | H072560534, H072680212 |
| 993471 | 1025009 | 31539 | H072560534, H072680212 |
| 923274 | 931183 | 7910 | H072560534, H072680212 |
| 895759 | 899486 | 3728 | H072560534, H072680212 |
| 1385404 | 1385410 | 7 | H072560534, H072680212 |
| 849764 | 851206 | 1443 | H072560534, H072680212 |
| 923274 | 948008 | 24735 | H091640624, H091720529, H100200320, H100200321, H100200319, H152780272, H152640286 |
| 2000302 | 2010965 | 10664 | EUL 43, EUL 37, EUL 44, EUL 45 |
| 294939 | 324541 | 29603 | H104780628, H100120270, H100280679, H114840679, H114820438, H114840676, H114840681, H114840678, H114840680, H114840677, H072300480, H072300481, H100180615, H100180614, H100280685, H072360603, H111920394, H100560549, H100280683, H100280682, H100560548, H111920402, H100180616, H104720329, H112000588, H072680210, H100180617, H100120260, H104780627, H120680630, H072680211, H104780626, H111920400, H113440613, H113440616, H113440614, H113580550, H113440612, H113440615, H113580549, H072560534, H072680212, H072680213, H111920398, H072360604, H111920404, H115180236, H115260949, H091640624, H091720529, H100200320, H100200321, H100200319, H152780272, H152640286 |
| 1182750 | 1192695 | 9946 | H104780628, H100120270, H100280679, H114840679, H114820438, H114840676, H114840681, H114840678, H114840680, H114840677, H072300480, H072300481, H100180615, H100180614, H100280685, H072360603, H111920394, H100560549, H100280683, H100280682, H100560548, H111920402, H100180616, H104720329, H112000588, H072680210, H100180617, H100120260, H104780627, H120680630, H072680211, H104780626, H111920400, H113440613, H113440616, H113440614, H113580550, H113440612, H113440615, H113580549, H072560534, H072680212, H072680213, H111920398, H072360604, H111920404, H115180236, H115260949, H091640624, H091720529, H100200320, H100200321, H100200319, H152780272, H152640286 |
| 149243 | 170570 | 21328 | H104780628, H100120270, H100280679, H114840679, H114820438, H114840676, H114840681, H114840678, H114840680, H114840677, H072300480, H072300481, H100180615, H100180614, H100280685, H072360603, H111920394, H100560549, H100280683, H100280682, H100560548, H111920402, H100180616, H104720329, H112000588, H072680210, H100180617, H100120260, H104780627, H120680630, H072680211, H104780626, H111920400, H113440613, H113440616, H113440614, H113580550, H113440612, H113440615, H113580549, H072560534, H072680212, H072680213, H111920398, H072360604, H111920404, H115180236, H115260949, H091640624, H091720529, H100200320, H100200321, H100200319, H152780272, H152640286 |
| 355606 | 364999 | 9394 | H104780628, H100120270, H100280679, H114840679, H114820438, H114840676, H114840681, H114840678, H114840680, H114840677, H072300480, H072300481, H100180615, H100180614, H100280685, H072360603, H111920394, H100560549, H100280683, H100280682, H100560548, H111920402, H100180616, H104720329, H112000588, H072680210, H100180617, H100120260, H104780627, H120680630, H072680211, H104780626, H111920400, H113440613, H113440616, H113440614, H113580550, H113440612, H113440615, H113580549, H072560534, H072680212, H072680213, H111920398, H072360604, H111920404, H115180236, H115260949, H091640624, H091720529, H100200320, H100200321, H100200319, H152780272, H152640286 |
| 1990908 | 2015184 | 24277 | H104780628, H100120270, H100280679, H114840679, H114820438, H114840676, H114840681, H114840678, H114840680, H114840677, H072300480, H072300481, H100180615, H100180614, H100280685, H072360603, H111920394, H100560549, H100280683, H100280682, H100560548, H111920402, H100180616, H104720329, H112000588, H072680210, H100180617, H100120260, H104780627, H120680630, H072680211, H104780626, H111920400, H113440613, H113440616, H113440614, H113580550, H113440612, H113440615, H113580549, H072560534, H072680212, H072680213, H111920398, H072360604, H111920404, H115180236, H115260949, H091640624, H091720529, H100200320, H100200321, H100200319, H152780272, H152640286 |
| 26366 | 41443 | 15078 | H104780628, H100120270, H100280679, H114840679, H114820438, H114840676, H114840681, H114840678, H114840680, H114840677, H072300480, H072300481, H100180615, H100180614, H100280685, H072360603, H111920394, H100560549, H100280683, H100280682, H100560548, H111920402, H100180616, H104720329, H112000588, H072680210, H100180617, H100120260, H104780627, H120680630, H072680211, H104780626, H111920400, H113440613, H113440616, H113440614, H113580550, H113440612, H113440615, H113580549, H072560534, H072680212, H072680213, H111920398, H072360604, H111920404, H115180236, H115260949, H091640624, H091720529, H100200320, H100200321, H100200319, H152780272, H152640286 |
| 3478376 | 3498139 | 19764 | H104780628, H100120270, H100280679, H114840679, H114820438, H114840676, H114840681, H114840678, H114840680, H114840677, H072300480, H072300481, H100180615, H100180614, H100280685, H072360603, H111920394, H100560549, H100280683, H100280682, H100560548, H111920402, H100180616, H104720329, H112000588, H072680210, H100180617, H100120260, H104780627, H120680630, H072680211, H104780626, H111920400, H113440613, H113440616, H113440614, H113580550, H113440612, H113440615, H113580549, H072560534, H072680212, H072680213, H111920398, H072360604, H111920404, H115180236, H115260949, H091640624, H091720529, H100200320, H100200321, H100200319, H152780272, H152640286 |
| 3441748 | 3459558 | 17811 | H104780628, H100120270, H100280679, H114840679, H114820438, H114840676, H114840681, H114840678, H114840680, H114840677, H072300480, H072300481, H100180615, H100180614, H100280685, H072360603, H111920394, H100560549, H100280683, H100280682, H100560548, H111920402, H100180616, H104720329, H112000588, H072680210, H100180617, H100120260, H104780627, H120680630, H072680211, H104780626, H111920400, H113440613, H113440616, H113440614, H113580550, H113440612, H113440615, H113580549, H072560534, H072680212, H072680213, H111920398, H072360604, H111920404, H115180236, H115260949, H091640624, H091720529, H100200320, H100200321, H100200319, H152780272, H152640286 |
| 1661334 | 1662527 | 1194 | H104780628, H100120270, H100280679, H114840679, H114820438, H114840676, H114840681, H114840678, H114840680, H114840677, H072300480, H072300481, H100180615, H100180614, H100280685, H072360603, H111920394, H100560549, H100280683, H100280682, H100560548, H111920402, H100180616, H104720329, H112000588, H072680210, H100180617, H100120260, H104780627, H120680630, H072680211, H104780626, H111920400, H113440613, H113440616, H113440614, H113580550, H113440612, H113440615, H113580549, H072560534, H072680212, H072680213, H111920398, H072360604, H111920404, H115180236, H115260949, H091640624, H091720529, H100200320, H100200321, H100200319, H152780272, H152640286 |
| 1814981 | 1834592 | 19612 | H104780628, H100120270, H100280679, H114840679, H114820438, H114840676, H114840681, H114840678, H114840680, H114840677, H072300480, H072300481, H100180615, H100180614, H100280685, H072360603, H111920394, H100560549, H100280683, H100280682, H100560548, H111920402, H100180616, H104720329, H112000588, H072680210, H100180617, H100120260, H104780627, H120680630, H072680211, H104780626, H111920400, H113440613, H113440616, H113440614, H113580550, H113440612, H113440615, H113580549, H072560534, H072680212, H072680213, H111920398, H072360604, H111920404, H115180236, H115260949, H091640624, H091720529, H100200320, H100200321, H100200319, H152780272, H152640286 |
| 1992558 | 2001487 | 8930 | H124240908, H124600775, H103120165, H103340763, EUL 17, H072740379, EUL 109, EUL 104, LP22_Sweden, LP23_Sweden, LP21_Sweden, EUL 108, HL04163014, Wien_47-14, HL00364001, Paris, HL 0101 3003, HL 0102 3034, HL 0102 3035, Paris_2001_I_n2, HL 0051 1015, HL 0131 3039, LG 0713 5006, LG 0713 5007, EUL 3, EUL 10, EUL 9, LG 0940 4015, EUL 42, HL 0230 4015, LG 1020 3012, LG 1019 1002, HL 0311 1005, LG 1101 1012, LG 1105 4025, LG 0919 2006, HL 0337 3012, LG 0725 3019, LG 0725 3022, LG 1427 4010, LG 1427 4009, LG 0918 2002, LG 0918 2005, LG 1416 4007, LG 1416 4008, ID_1828, E21203, EUL 43, EUL 37, EUL 44, EUL 45, H104780628, H100120270, H100280679, H114840679, H114820438, H114840676, H114840681, H114840678, H114840680, H114840677, H072300480, H072300481, H100180615, H100180614, H100280685, H072360603, H111920394, H100560549, H100280683, H100280682, H100560548, H111920402, H100180616, H104720329, H112000588, H072680210, H100180617, H100120260, H104780627, H120680630, H072680211, H104780626, H111920400, H113440613, H113440616, H113440614, H113580550, H113440612, H113440615, H113580549, H072560534, H072680212, H072680213, H111920398, H072360604, H111920404, H115180236, H115260949, H091640624, H091720529, H100200320, H100200321, H100200319, H152780272, H152640286 |
| 3061410 | 3069294 | 7885 | Lp-120, Lp-119, Lp-121, Lp-122, Lp-285, Lp-283, Lp-286-1, NIIB223, NIIB225, NIIB80, OLDA1, EUL 53, ID_1690, EUL 62, EUL 67, EUL 58, EUL 55, LP43 |
| 2319666 | 2324386 | 4721 | EUL 1, LT40-04, EUL 60, ID_891, ID_598, ID_2947, H124240908, H124600775, H103120165, H103340763, EUL 17, H072740379, EUL 109, EUL 104, LP22_Sweden, LP23_Sweden, LP21_Sweden, EUL 108, HL 0416 3014, Wien_47-14, HL 0036 4001, Paris, HL 0101 3003, HL 0102 3034, HL 0102 3035, Paris_2001_I_n2, HL 0051 1015, HL 0131 3039, LG 0713 5006, LG 0713 5007, EUL 3, EUL 10, EUL 9, LG 0940 4015, EUL 42, HL 0230 4015, LG 1020 3012, LG 1019 1002, HL 0311 1005, LG 1101 1012, LG 1105 4025, LG 0919 2006, HL 0337 3012, LG 0725 3019, LG 0725 3022, LG 1427 4010, LG 1427 4009, LG 0918 2002, LG 0918 2005, LG 1416 4007, LG 1416 4008, ID_1828, E21203, EUL 43, EUL 37, EUL 44, EUL 45, H104780628, H100120270, H100280679, H114840679, H114820438, H114840676, H114840681, H114840678, H114840680, H114840677, H072300480, H072300481, H100180615, H100180614, H100280685, H072360603, H111920394, H100560549, H100280683, H100280682, H100560548, H111920402, H100180616, H104720329, H112000588, H072680210, H100180617, H100120260, H104780627, H120680630, H072680211, H104780626, H111920400, H113440613, H113440616, H113440614, H113580550, H113440612, H113440615, H113580549, H072560534, H072680212, H072680213, H111920398, H072360604, H111920404, H115180236, H115260949, H091640624, H091720529, H100200320, H100200321, H100200319, H152780272, H152640286 |
| 2014953 | 2018845 | 3893 | EUL 1, LT40-04, EUL 60, ID_891, ID_598, ID_2947, H124240908, H124600775, H103120165, H103340763, EUL 17, H072740379, EUL 109, EUL 104, LP22_Sweden, LP23_Sweden, LP21_Sweden, EUL 108, HL 0416 3014, Wien_47-14, HL 0036 4001, Paris, HL 0101 3003, HL 0102 3034, HL 0102 3035, Paris_2001_I_n2, HL 0051 1015, HL 0131 3039, LG 0713 5006, LG 0713 5007, EUL 3, EUL 10, EUL 9, LG 0940 4015, EUL 42, HL 0230 4015, LG 1020 3012, LG 1019 1002, HL 0311 1005, LG 1101 1012, LG 1105 4025, LG 0919 2006, HL 0337 3012, LG 0725 3019, LG 0725 3022, LG 1427 4010, LG 1427 4009, LG 0918 2002, LG 0918 2005, LG 1416 4007, LG 1416 4008, ID_1828, E21203, EUL 43, EUL 37, EUL 44, EUL 45, H104780628, H100120270, H100280679, H114840679, H114820438, H114840676, H114840681, H114840678, H114840680, H114840677, H072300480, H072300481, H100180615, H100180614, H100280685, H072360603, H111920394, H100560549, H100280683, H100280682, H100560548, H111920402, H100180616, H104720329, H112000588, H072680210, H100180617, H100120260, H104780627, H120680630, H072680211, H104780626, H111920400, H113440613, H113440616, H113440614, H113580550, H113440612, H113440615, H113580549, H072560534, H072680212, H072680213, H111920398, H072360604, H111920404, H115180236, H115260949, H091640624, H091720529, H100200320, H100200321, H100200319, H152780272, H152640286 |
| 2873572 | 2942044 | 68473 | EUL 21, EUL 14, EUL 13, EUL 16 |
| 1986118 | 2005626 | 19509 | EUL 21, EUL 14, EUL 13, EUL 16 |
| 2125861 | 2148867 | 23007 | EUL 21, EUL 14, EUL 13, EUL 16 |
| 2964210 | 2994118 | 29909 | EUL 21, EUL 14, EUL 13, EUL 16 |
| 401143 | 432534 | 31392 | EUL 21, EUL 14, EUL 13, EUL 16 |
| 1315589 | 1341685 | 26097 | EUL 21, EUL 14, EUL 13, EUL 16 |
| 2795686 | 2817256 | 21571 | EUL 21, EUL 14, EUL 13, EUL 16 |
| 68787 | 74429 | 5643 | EUL 21, EUL 14, EUL 13, EUL 16 |
| 2851866 | 2868994 | 17129 | EUL 21, EUL 14, EUL 13, EUL 16 |
| 1843392 | 1853272 | 9881 | EUL 21, EUL 14, EUL 13, EUL 16 |
| 3017846 | 3052614 | 34769 | EUL 21, EUL 14, EUL 13, EUL 16 |
| 2428004 | 2439942 | 11939 | EUL 21, EUL 14, EUL 13, EUL 16 |
| 1509886 | 1513332 | 3447 | EUL 21, EUL 14, EUL 13, EUL 16 |
| 1871371 | 1880112 | 8742 | EUL 21, EUL 14, EUL 13, EUL 16 |
| 2942651 | 2950814 | 8164 | EUL 21, EUL 14, EUL 13, EUL 16 |
| 1972681 | 1976119 | 3439 | EUL 21, EUL 14, EUL 13, EUL 16 |
| 1589801 | 1595969 | 6169 | EUL 21, EUL 14, EUL 13, EUL 16 |
| 437938 | 443814 | 5877 | EUL 21, EUL 14, EUL 13, EUL 16 |
| 2617972 | 2620919 | 2948 | EUL 21, EUL 14, EUL 13, EUL 16 |
| 2518655 | 2518982 | 328 | EUL 21, EUL 14, EUL 13, EUL 16 |
| 75660 | 76221 | 562 | Lp-120, Lp-119, Lp-121, Lp-122, Lp-285, Lp-283, Lp-286-1, NIIB223, NIIB225, NIIB80, OLDA1, EUL 53, ID_1690, EUL 62, EUL 67, EUL 58, EUL 55, LP43, EUL 1, LT40-04, EUL 60, ID_891, ID_598, ID_2947, H124240908, H124600775, H103120165, H103340763, EUL 17, H072740379, EUL 109, EUL 104, LP22_Sweden, LP23_Sweden, LP21_Sweden, EUL 108, HL 0416 3014, Wien_47-14, HL 0036 4001, Paris, HL 0101 3003, HL 0102 3034, HL 0102 3035, Paris_2001_I_n2, HL 0051 1015, HL 0131 3039, LG 0713 5006, LG 0713 5007, EUL 3, EUL 10, EUL 9, LG 0940 4015, EUL 42, HL 0230 4015, LG 1020 3012, LG 1019 1002, HL 0311 1005, LG 1101 1012, LG 1105 4025, LG 0919 2006, HL 0337 3012, LG 0725 3019, LG 0725 3022, LG 1427 4010, LG 1427 4009, LG 0918 2002, LG 0918 2005, LG 1416 4007, LG 1416 4008, ID_1828, E21203, EUL 43, EUL 37, EUL 44, EUL 45, H104780628, H100120270, H100280679, H114840679, H114820438, H114840676, H114840681, H114840678, H114840680, H114840677, H072300480, H072300481, H100180615, H100180614, H100280685, H072360603, H111920394, H100560549, H100280683, H100280682, H100560548, H111920402, H100180616, H104720329, H112000588, H072680210, H100180617, H100120260, H104780627, H120680630, H072680211, H104780626, H111920400, H113440613, H113440616, H113440614, H113580550, H113440612, H113440615, H113580549, H072560534, H072680212, H072680213, H111920398, H072360604, H111920404, H115180236, H115260949, H091640624, H091720529, H100200320, H100200321, H100200319, H152780272, H152640286 |
| 3071565 | 3142243 | 70679 | ID_2948 |
| 2972971 | 2993161 | 20191 | ID_2948 |
| 3003881 | 3016674 | 12794 | ID_2948 |
| 3260124 | 3272911 | 12788 | ID_2948 |
| 150306 | 157405 | 7100 | ID_2948 |
| 2928006 | 2929589 | 1584 | ID_2948 |
| 923274 | 951358 | 28085 | 2735, H101460286, H101740836, H074360702, H134660746, H073300079, H073300077, H073360657, H092520167, H092620872, H103620682, H102860194, Lp-284, Lp-282-1, EUL 90, EUL 82, EUL 85, EUL 88, EUL 95, EUL 94, EUL 93, EUL 84, H085060063, H084800579 |
| 1648686 | 1675289 | 26604 | 2735, H101460286, H101740836, H074360702, H134660746, H073300079, H073300077, H073360657, H092520167, H092620872, H103620682, H102860194, Lp-284, Lp-282-1, EUL 90, EUL 82, EUL 85, EUL 88, EUL 95, EUL 94, EUL 93, EUL 84, H085060063, H084800579 |
| 1992350 | 2001487 | 9138 | 2735, H101460286, H101740836, H074360702, H134660746, H073300079, H073300077, H073360657, H092520167, H092620872, H103620682, H102860194, Lp-284, Lp-282-1, EUL 90, EUL 82, EUL 85, EUL 88, EUL 95, EUL 94, EUL 93, EUL 84, H085060063, H084800579 |
| 895759 | 899486 | 3728 | 2735, H101460286, H101740836, H074360702, H134660746, H073300079, H073300077, H073360657, H092520167, H092620872, H103620682, H102860194, Lp-284, Lp-282-1, EUL 90, EUL 82, EUL 85, EUL 88, EUL 95, EUL 94, EUL 93, EUL 84, H085060063, H084800579 |
| 68053 | 74429 | 6377 | ID_2948, EUL 21, EUL 14, EUL 13, EUL 16, Lp-120, Lp-119, Lp-121, Lp-122, Lp-285, Lp-283, Lp-286-1, NIIB223, NIIB225, NIIB80, OLDA1, EUL 53, ID_1690, EUL 62, EUL 67, EUL 58, EUL 55, LP43, EUL 1, LT40-04, EUL 60, ID_891, ID_598, ID_2947, H124240908, H124600775, H103120165, H103340763, EUL 17, H072740379, EUL 109, EUL 104, LP22_Sweden, LP23_Sweden, LP21_Sweden, EUL 108, HL 0416 3014, Wien_47-14, HL 0036 4001, Paris, HL 0101 3003, HL 0102 3034, HL 0102 3035, Paris_2001_I_n2, HL 0051 1015, HL 0131 3039, LG 0713 5006, LG 0713 5007, EUL 3, EUL 10, EUL 9, LG 0940 4015, EUL 42, HL 0230 4015, LG 1020 3012, LG 1019 1002, HL 0311 1005, LG 1101 1012, LG 1105 4025, LG 0919 2006, HL 0337 3012, LG 0725 3019, LG 0725 3022, LG 1427 4010, LG 1427 4009, LG 0918 2002, LG 0918 2005, LG 1416 4007, LG 1416 4008, ID 1828, E21203, EUL 43, EUL 37, EUL 44, EUL 45, H104780628, H100120270, H100280679, H114840679, H114820438, H114840676, H114840681, H114840678, H114840680, H114840677, H072300480, H072300481, H100180615, H100180614, H100280685, H072360603, H111920394, H100560549, H100280683, H100280682, H100560548, H111920402, H100180616, H104720329, H112000588, H072680210, H100180617, H100120260, H104780627, H120680630, H072680211, H104780626, H111920400, H113440613, H113440616, H113440614, H113580550, H113440612, H113440615, H113580549, H072560534, H072680212, H072680213, H111920398, H072360604, H111920404, H115180236, H115260949, H091640624, H091720529, H100200320, H100200321, H100200319, H152780272, H152640286 |
| 2055666 | 2061785 | 6120 | ID_2948, EUL 21, EUL 14, EUL 13, EUL 16, Lp-120, Lp-119, Lp-121, Lp-122, Lp-285, Lp-283, Lp-286-1, NIIB223, NIIB225, NIIB80, OLDA1, EUL 53, ID_1690, EUL 62, EUL 67, EUL 58, EUL 55, LP43, EUL 1, LT40-04, EUL 60, ID_891, ID_598, ID_2947, H124240908, H124600775, H103120165, H103340763, EUL 17, H072740379, EUL 109, EUL 104, LP22_Sweden, LP23_Sweden, LP21_Sweden, EUL 108, HL 0416 3014, Wien_47-14, HL 0036 4001, Paris, HL 0101 3003, HL 0102 3034, HL 0102 3035, Paris_2001_I_n2, HL 0051 1015, HL 0131 3039, LG 0713 5006, LG 0713 5007, EUL 3, EUL 10, EUL 9, LG 0940 4015, EUL 42, HL 0230 4015, LG 1020 3012, LG 1019 1002, HL 0311 1005, LG 1101 1012, LG 1105 4025, LG 0919 2006, HL 0337 3012, LG 0725 3019, LG 0725 3022, LG 1427 4010, LG 1427 4009, LG 0918 2002, LG 0918 2005, LG 1416 4007, LG 1416 4008, ID 1828, E21203, EUL 43, EUL 37, EUL 44, EUL 45, H104780628, H100120270, H100280679, H114840679, H114820438, H114840676, H114840681, H114840678, H114840680, H114840677, H072300480, H072300481, H100180615, H100180614, H100280685, H072360603, H111920394, H100560549, H100280683, H100280682, H100560548, H111920402, H100180616, H104720329, H112000588, H072680210, H100180617, H100120260, H104780627, H120680630, H072680211, H104780626, H111920400, H113440613, H113440616, H113440614, H113580550, H113440612, H113440615, H113580549, H072560534, H072680212, H072680213, H111920398, H072360604, H111920404, H115180236, H115260949, H091640624, H091720529, H100200320, H100200321, H100200319, H152780272, H152640286 |
| 2072723 | 2076994 | 4272 | ID_2948, EUL 21, EUL 14, EUL 13, EUL 16, Lp-120, Lp-119, Lp-121, Lp-122, Lp-285, Lp-283, Lp-286-1, NIIB223, NIIB225, NIIB80, OLDA1, EUL 53, ID_1690, EUL 62, EUL 67, EUL 58, EUL 55, LP43, EUL 1, LT40-04, EUL 60, ID_891, ID_598, ID_2947, H124240908, H124600775, H103120165, H103340763, EUL 17, H072740379, EUL 109, EUL 104, LP22_Sweden, LP23_Sweden, LP21_Sweden, EUL 108, HL 0416 3014, Wien_47-14, HL 0036 4001, Paris, HL 0101 3003, HL 0102 3034, HL 0102 3035, Paris_2001_I_n2, HL 0051 1015, HL 0131 3039, LG 0713 5006, LG 0713 5007, EUL 3, EUL 10, EUL 9, LG 0940 4015, EUL 42, HL 0230 4015, LG 1020 3012, LG 1019 1002, HL 0311 1005, LG 1101 1012, LG 1105 4025, LG 0919 2006, HL 0337 3012, LG 0725 3019, LG 0725 3022, LG 1427 4010, LG 1427 4009, LG 0918 2002, LG 0918 2005, LG 1416 4007, LG 1416 4008, ID 1828, E21203, EUL 43, EUL 37, EUL 44, EUL 45, H104780628, H100120270, H100280679, H114840679, H114820438, H114840676, H114840681, H114840678, H114840680, H114840677, H072300480, H072300481, H100180615, H100180614, H100280685, H072360603, H111920394, H100560549, H100280683, H100280682, H100560548, H111920402, H100180616, H104720329, H112000588, H072680210, H100180617, H100120260, H104780627, H120680630, H072680211, H104780626, H111920400, H113440613, H113440616, H113440614, H113580550, H113440612, H113440615, H113580549, H072560534, H072680212, H072680213, H111920398, H072360604, H111920404, H115180236, H115260949, H091640624, H091720529, H100200320, H100200321, H100200319, H152780272, H152640286 |
| 2084600 | 2094628 | 10029 | ID_2948, EUL 21, EUL 14, EUL 13, EUL 16, Lp-120, Lp-119, Lp-121, Lp-122, Lp-285, Lp-283, Lp-286-1, NIIB223, NIIB225, NIIB80, OLDA1, EUL 53, ID_1690, EUL 62, EUL 67, EUL 58, EUL 55, LP43, EUL 1, LT40-04, EUL 60, ID_891, ID_598, ID_2947, H124240908, H124600775, H103120165, H103340763, EUL 17, H072740379, EUL 109, EUL 104, LP22_Sweden, LP23_Sweden, LP21_Sweden, EUL 108, HL 0416 3014, Wien_47-14, HL 0036 4001, Paris, HL 0101 3003, HL 0102 3034, HL 0102 3035, Paris_2001_I_n2, HL 0051 1015, HL 0131 3039, LG 0713 5006, LG 0713 5007, EUL 3, EUL 10, EUL 9, LG 0940 4015, EUL 42, HL 0230 4015, LG 1020 3012, LG 1019 1002, HL 0311 1005, LG 1101 1012, LG 1105 4025, LG 0919 2006, HL 0337 3012, LG 0725 3019, LG 0725 3022, LG 1427 4010, LG 1427 4009, LG 0918 2002, LG 0918 2005, LG 1416 4007, LG 1416 4008, ID 1828, E21203, EUL 43, EUL 37, EUL 44, EUL 45, H104780628, H100120270, H100280679, H114840679, H114820438, H114840676, H114840681, H114840678, H114840680, H114840677, H072300480, H072300481, H100180615, H100180614, H100280685, H072360603, H111920394, H100560549, H100280683, H100280682, H100560548, H111920402, H100180616, H104720329, H112000588, H072680210, H100180617, H100120260, H104780627, H120680630, H072680211, H104780626, H111920400, H113440613, H113440616, H113440614, H113580550, H113440612, H113440615, H113580549, H072560534, H072680212, H072680213, H111920398, H072360604, H111920404, H115180236, H115260949, H091640624, H091720529, H100200320, H100200321, H100200319, H152780272, H152640286 |
| 467796 | 476192 | 8397 | ID_2948, EUL 21, EUL 14, EUL 13, EUL 16, Lp-120, Lp-119, Lp-121, Lp-122, Lp-285, Lp-283, Lp-286-1, NIIB223, NIIB225, NIIB80, OLDA1, EUL 53, ID_1690, EUL 62, EUL 67, EUL 58, EUL 55, LP43, EUL 1, LT40-04, EUL 60, ID_891, ID_598, ID_2947, H124240908, H124600775, H103120165, H103340763, EUL 17, H072740379, EUL 109, EUL 104, LP22_Sweden, LP23_Sweden, LP21_Sweden, EUL 108, HL 0416 3014, Wien_47-14, HL 0036 4001, Paris, HL 0101 3003, HL 0102 3034, HL 0102 3035, Paris_2001_I_n2, HL 0051 1015, HL 0131 3039, LG 0713 5006, LG 0713 5007, EUL 3, EUL 10, EUL 9, LG 0940 4015, EUL 42, HL 0230 4015, LG 1020 3012, LG 1019 1002, HL 0311 1005, LG 1101 1012, LG 1105 4025, LG 0919 2006, HL 0337 3012, LG 0725 3019, LG 0725 3022, LG 1427 4010, LG 1427 4009, LG 0918 2002, LG 0918 2005, LG 1416 4007, LG 1416 4008, ID 1828, E21203, EUL 43, EUL 37, EUL 44, EUL 45, H104780628, H100120270, H100280679, H114840679, H114820438, H114840676, H114840681, H114840678, H114840680, H114840677, H072300480, H072300481, H100180615, H100180614, H100280685, H072360603, H111920394, H100560549, H100280683, H100280682, H100560548, H111920402, H100180616, H104720329, H112000588, H072680210, H100180617, H100120260, H104780627, H120680630, H072680211, H104780626, H111920400, H113440613, H113440616, H113440614, H113580550, H113440612, H113440615, H113580549, H072560534, H072680212, H072680213, H111920398, H072360604, H111920404, H115180236, H115260949, H091640624, H091720529, H100200320, H100200321, H100200319, H152780272, H152640286 |
| 2928006 | 2929589 | 1584 | ID_2948, EUL 21, EUL 14, EUL 13, EUL 16, Lp-120, Lp-119, Lp-121, Lp-122, Lp-285, Lp-283, Lp-286-1, NIIB223, NIIB225, NIIB80, OLDA1, EUL 53, ID_1690, EUL 62, EUL 67, EUL 58, EUL 55, LP43, EUL 1, LT40-04, EUL 60, ID_891, ID_598, ID_2947, H124240908, H124600775, H103120165, H103340763, EUL 17, H072740379, EUL 109, EUL 104, LP22_Sweden, LP23_Sweden, LP21_Sweden, EUL 108, HL 0416 3014, Wien_47-14, HL 0036 4001, Paris, HL 0101 3003, HL 0102 3034, HL 0102 3035, Paris_2001_I_n2, HL 0051 1015, HL 0131 3039, LG 0713 5006, LG 0713 5007, EUL 3, EUL 10, EUL 9, LG 0940 4015, EUL 42, HL 0230 4015, LG 1020 3012, LG 1019 1002, HL 0311 1005, LG 1101 1012, LG 1105 4025, LG 0919 2006, HL 0337 3012, LG 0725 3019, LG 0725 3022, LG 1427 4010, LG 1427 4009, LG 0918 2002, LG 0918 2005, LG 1416 4007, LG 1416 4008, ID 1828, E21203, EUL 43, EUL 37, EUL 44, EUL 45, H104780628, H100120270, H100280679, H114840679, H114820438, H114840676, H114840681, H114840678, H114840680, H114840677, H072300480, H072300481, H100180615, H100180614, H100280685, H072360603, H111920394, H100560549, H100280683, H100280682, H100560548, H111920402, H100180616, H104720329, H112000588, H072680210, H100180617, H100120260, H104780627, H120680630, H072680211, H104780626, H111920400, H113440613, H113440616, H113440614, H113580550, H113440612, H113440615, H113580549, H072560534, H072680212, H072680213, H111920398, H072360604, H111920404, H115180236, H115260949, H091640624, H091720529, H100200320, H100200321, H100200319, H152780272, H152640286 |
| 2503938 | 2533058 | 29121 | L00-549, LG 1118 1044, EUL 119, EUL 114, EUL 113, LG 1014 3009, EUL 110, HL 0701 3004, ID_6885, ATCC 35289, EUL 46, LG 1139 1124, LP15, LP14, LP37, LP31, LP30, LP40, LP41, LP45, LP46, LP20, LP02, LP04, LP03, LP01, LP05, LP24, LP10, LP27, LP26, LP28, LP19, LP16, LP18, LP25, LP39, LP38, LP11, LP34, LP36, LP29, LP35, LP12, LP17, LP09, LP32, LP21, LP44, LP06, LP33, LP23, LP48, LP47, LP07, LP08, ID_2041, ID_1688, ID_747970, Lp-2002694p8, Lp-56207 |
| 2592582 | 2599836 | 7255 | L00-549, LG 1118 1044, EUL 119, EUL 114, EUL 113, LG 1014 3009, EUL 110, HL 0701 3004, ID_6885, ATCC 35289, EUL 46, LG 1139 1124, LP15, LP14, LP37, LP31, LP30, LP40, LP41, LP45, LP46, LP20, LP02, LP04, LP03, LP01, LP05, LP24, LP10, LP27, LP26, LP28, LP19, LP16, LP18, LP25, LP39, LP38, LP11, LP34, LP36, LP29, LP35, LP12, LP17, LP09, LP32, LP21, LP44, LP06, LP33, LP23, LP48, LP47, LP07, LP08, ID_2041, ID_1688, ID_747970, Lp-2002694p8, Lp-56207 |
| 2552448 | 2558952 | 6505 | L00-549, LG 1118 1044, EUL 119, EUL 114, EUL 113, LG 1014 3009, EUL 110, HL 0701 3004, ID_6885, ATCC 35289, EUL 46, LG 1139 1124, LP15, LP14, LP37, LP31, LP30, LP40, LP41, LP45, LP46, LP20, LP02, LP04, LP03, LP01, LP05, LP24, LP10, LP27, LP26, LP28, LP19, LP16, LP18, LP25, LP39, LP38, LP11, LP34, LP36, LP29, LP35, LP12, LP17, LP09, LP32, LP21, LP44, LP06, LP33, LP23, LP48, LP47, LP07, LP08, ID_2041, ID_1688, ID_747970, Lp-2002694p8, Lp-56207 |
| 2672086 | 2687329 | 15244 | HL 0051 4008, LG 0713 5008, HL 0131 3038 |
| 1238465 | 1283775 | 45311 | HL 0051 4008, LG 0713 5008, HL 0131 3038 |
| 392607 | 412712 | 20106 | HL 0051 4008, LG 0713 5008, HL 0131 3038 |
| 293793 | 316313 | 22521 | HL 0051 4008, LG 0713 5008, HL 0131 3038 |
| 1679301 | 1685488 | 6188 | HL 0051 4008, LG 0713 5008, HL 0131 3038 |
| 3036187 | 3071400 | 35214 | HL 0051 4008, LG 0713 5008, HL 0131 3038 |
| 2711445 | 2712588 | 1144 | HL 0051 4008, LG 0713 5008, HL 0131 3038 |
| 1625198 | 1662202 | 37005 | HL 0051 4008, LG 0713 5008, HL 0131 3038 |
| 264765 | 287566 | 22802 | HL 0051 4008, LG 0713 5008, HL 0131 3038 |
| 1842409 | 1846014 | 3606 | HL 0051 4008, LG 0713 5008, HL 0131 3038 |
| 2074098 | 2109059 | 34962 | HL 0051 4008, LG 0713 5008, HL 0131 3038 |
| 2055975 | 2061232 | 5258 | HL 0051 4008, LG 0713 5008, HL 0131 3038 |
| 2303945 | 2321603 | 17659 | HL 0051 4008, LG 0713 5008, HL 0131 3038 |
| 1306771 | 1307587 | 817 | HL 0051 4008, LG 0713 5008, HL 0131 3038 |
| 365778 | 372180 | 6403 | HL 0051 4008, LG 0713 5008, HL 0131 3038 |
| 1408337 | 1413362 | 5026 | HL 0051 4008, LG 0713 5008, HL 0131 3038 |
| 1898083 | 1911795 | 13713 | HL 0051 4008, LG 0713 5008, HL 0131 3038 |
| 1031495 | 1034112 | 2618 | HL 0051 4008, LG 0713 5008, HL 0131 3038 |
| 1348007 | 1355606 | 7600 | HL 0051 4008, LG 0713 5008, HL 0131 3038 |
| 1212081 | 1217990 | 5910 | HL 0051 4008, LG 0713 5008, HL 0131 3038 |
| 1327893 | 1341686 | 13794 | HL 0051 4008, LG 0713 5008, HL 0131 3038 |
| 980725 | 988219 | 7495 | HL 0051 4008, LG 0713 5008, HL 0131 3038 |
| 1873526 | 1884185 | 10660 | HL 0051 4008, LG 0713 5008, HL 0131 3038 |
| 2641726 | 2645484 | 3759 | HL 0051 4008, LG 0713 5008, HL 0131 3038 |
| 2117031 | 2129700 | 12670 | HL 0051 4008, LG 0713 5008, HL 0131 3038 |
| 1594962 | 1616765 | 21804 | HL 0051 4008, LG 0713 5008, HL 0131 3038 |
| 1130915 | 1141928 | 11014 | HL 0051 4008, LG 0713 5008, HL 0131 3038 |
| 1942697 | 1946973 | 4277 | HL 0051 4008, LG 0713 5008, HL 0131 3038 |
| 1041518 | 1051958 | 10441 | HL 0051 4008, LG 0713 5008, HL 0131 3038 |
| 1807091 | 1815858 | 8768 | HL 0051 4008, LG 0713 5008, HL 0131 3038 |
| 1669220 | 1675512 | 6293 | HL 0051 4008, LG 0713 5008, HL 0131 3038 |
| 1700666 | 1707467 | 6802 | HL 0051 4008, LG 0713 5008, HL 0131 3038 |
| 1823545 | 1835337 | 11793 | HL 0051 4008, LG 0713 5008, HL 0131 3038 |
| 1286406 | 1292384 | 5979 | HL 0051 4008, LG 0713 5008, HL 0131 3038 |
| 288176 | 293515 | 5340 | HL 0051 4008, LG 0713 5008, HL 0131 3038 |
| 3267597 | 3268816 | 1220 | HL 0051 4008, LG 0713 5008, HL 0131 3038 |
| 1015923 | 1024718 | 8796 | HL 0051 4008, LG 0713 5008, HL 0131 3038 |
| 2031076 | 2044854 | 13779 | HL 0051 4008, LG 0713 5008, HL 0131 3038 |
| 1977731 | 1981242 | 3512 | HL 0051 4008, LG 0713 5008, HL 0131 3038 |
| 941422 | 944744 | 3323 | EUL 117, L3415-03, L3386-03, H034800423 |
| 1443483 | 1481944 | 38462 | EUL 117, L3415-03, L3386-03, H034800423 |
| 1490300 | 1520516 | 30217 | EUL 117, L3415-03, L3386-03, H034800423 |
| 3299584 | 3315343 | 15760 | EUL 117, L3415-03, L3386-03, H034800423 |
| 787507 | 792757 | 5251 | EUL 117, L3415-03, L3386-03, H034800423 |
| 1573150 | 1584046 | 10897 | EUL 117, L3415-03, L3386-03, H034800423 |
| 1530132 | 1540935 | 10804 | EUL 117, L3415-03, L3386-03, H034800423 |
| 1725961 | 1732862 | 6902 | EUL 117, L3415-03, L3386-03, H034800423 |
| 2619241 | 2634290 | 15050 | EUL 157, Lp-032, L00-549, LG 1118 1044, EUL 119, EUL 114, EUL 113, LG 1014 3009, EUL 110, HL 0701 3004, ID_6885, ATCC 35289, EUL 46, LG 1139 1124, LP15, LP14, LP37, LP31, LP30, LP40, LP41, LP45, LP46, LP20, LP02, LP04, LP03, LP01, LP05, LP24, LP10, LP27, LP26, LP28, LP19, LP16, LP18, LP25, LP39, LP38, LP11, LP34, LP36, LP29, LP35, LP12, LP17, LP09, LP32, LP21, LP44, LP06, LP33, LP23, LP48, LP47, LP07, LP08, ID_2041, ID_1688, ID_747970, Lp-2002694p8, Lp-56207, 2735, H101460286, H101740836, H074360702, H134660746, H073300079, H073300077, H073360657, H092520167, H092620872, H103620682, H102860194, Lp-284, Lp-282-1, EUL 90, EUL 82, EUL 85, EUL 88, EUL 95, EUL 94, EUL 93, EUL 84, H085060063, H084800579, ID_2948, EUL 21, EUL 14, EUL 13, EUL 16, Lp-120, Lp-119, Lp-121, Lp-122, Lp-285, Lp-283, Lp-286-1, NIIB223, NIIB225, NIIB80, OLDA1, EUL 53, ID_1690, EUL 62, EUL 67, EUL 58, EUL 55, LP43, EUL 1, LT40-04, EUL 60, ID_891, ID_598, ID_2947, H124240908, H124600775, H103120165, H103340763, EUL 17, H072740379, EUL 109, EUL 104, LP22_Sweden, LP23_Sweden, LP21_Sweden, EUL 108, HL 0416 3014,Wien_47-14, HL 0036 4001, Paris, HL 0101 3003, HL 0102 3034, HL 0102 3035, Paris_2001_I_n2, HL 0051 1015, HL 0131 3039, LG 0713 5006, LG 0713 5007, EUL 3, EUL 10, EUL 9, LG 0940 4015, EUL 42, HL 0230 4015, LG 1020 3012, LG 1019 1002, HL 0311 1005, LG 1101 1012, LG 1105 4025, LG 0919 2006, HL 0337 3012, LG 0725 3019, LG 0725 3022, LG 1427 4010, LG 1427 4009, LG 0918 2002, LG 0918 2005, LG 1416 4007, LG 1416 4008, ID_1828, E21203, EUL 43, EUL 37, EUL 44, EUL 45, H104780628, H100120270, H100280679, H114840679, H114820438, H114840676, H114840681, H114840678, H114840680, H114840677, H072300480, H072300481, H100180615, H100180614, H100280685, H072360603, H111920394, H100560549, H100280683, H100280682, H100560548, H111920402, H100180616, H104720329, H112000588, H072680210, H100180617, H100120260, H104780627, H120680630, H072680211, H104780626, H111920400, H113440613, H113440616, H113440614, H113580550, H113440612, H113440615, H113580549, H072560534, H072680212, H072680213, H111920398, H072360604, H111920404, H115180236, H115260949, H091640624, H091720529, H100200320, H100200321, H100200319, H152780272, H152640286 |
| 1790015 | 1796290 | 6276 | EUL 157, Lp-032, L00-549, LG 1118 1044, EUL 119, EUL 114, EUL 113, LG 1014 3009, EUL 110, HL 0701 3004, ID_6885, ATCC 35289, EUL 46, LG 1139 1124, LP15, LP14, LP37, LP31, LP30, LP40, LP41, LP45, LP46, LP20, LP02, LP04, LP03, LP01, LP05, LP24, LP10, LP27, LP26, LP28, LP19, LP16, LP18, LP25, LP39, LP38, LP11, LP34, LP36, LP29, LP35, LP12, LP17, LP09, LP32, LP21, LP44, LP06, LP33, LP23, LP48, LP47, LP07, LP08, ID_2041, ID_1688, ID_747970, Lp-2002694p8, Lp-56207, 2735, H101460286, H101740836, H074360702, H134660746, H073300079, H073300077, H073360657, H092520167, H092620872, H103620682, H102860194, Lp-284, Lp-282-1, EUL 90, EUL 82, EUL 85, EUL 88, EUL 95, EUL 94, EUL 93, EUL 84, H085060063, H084800579, ID_2948, EUL 21, EUL 14, EUL 13, EUL 16, Lp-120, Lp-119, Lp-121, Lp-122, Lp-285, Lp-283, Lp-286-1, NIIB223, NIIB225, NIIB80, OLDA1, EUL 53, ID_1690, EUL 62, EUL 67, EUL 58, EUL 55, LP43, EUL 1, LT40-04, EUL 60, ID_891, ID_598, ID_2947, H124240908, H124600775, H103120165, H103340763, EUL 17, H072740379, EUL 109, EUL 104, LP22_Sweden, LP23_Sweden, LP21_Sweden, EUL 108, HL 0416 3014,Wien_47-14, HL 0036 4001, Paris, HL 0101 3003, HL 0102 3034, HL 0102 3035, Paris_2001_I_n2, HL 0051 1015, HL 0131 3039, LG 0713 5006, LG 0713 5007, EUL 3, EUL 10, EUL 9, LG 0940 4015, EUL 42, HL 0230 4015, LG 1020 3012, LG 1019 1002, HL 0311 1005, LG 1101 1012, LG 1105 4025, LG 0919 2006, HL 0337 3012, LG 0725 3019, LG 0725 3022, LG 1427 4010, LG 1427 4009, LG 0918 2002, LG 0918 2005, LG 1416 4007, LG 1416 4008, ID_1828, E21203, EUL 43, EUL 37, EUL 44, EUL 45, H104780628, H100120270, H100280679, H114840679, H114820438, H114840676, H114840681, H114840678, H114840680, H114840677, H072300480, H072300481, H100180615, H100180614, H100280685, H072360603, H111920394, H100560549, H100280683, H100280682, H100560548, H111920402, H100180616, H104720329, H112000588, H072680210, H100180617, H100120260, H104780627, H120680630, H072680211, H104780626, H111920400, H113440613, H113440616, H113440614, H113580550, H113440612, H113440615, H113580549, H072560534, H072680212, H072680213, H111920398, H072360604, H111920404, H115180236, H115260949, H091640624, H091720529, H100200320, H100200321, H100200319, H152780272, H152640286 |
| 2647068 | 2648366 | 1299 | EUL 157, Lp-032, L00-549, LG 1118 1044, EUL 119, EUL 114, EUL 113, LG 1014 3009, EUL 110, HL 0701 3004, ID_6885, ATCC 35289, EUL 46, LG 1139 1124, LP15, LP14, LP37, LP31, LP30, LP40, LP41, LP45, LP46, LP20, LP02, LP04, LP03, LP01, LP05, LP24, LP10, LP27, LP26, LP28, LP19, LP16, LP18, LP25, LP39, LP38, LP11, LP34, LP36, LP29, LP35, LP12, LP17, LP09, LP32, LP21, LP44, LP06, LP33, LP23, LP48, LP47, LP07, LP08, ID_2041, ID_1688, ID_747970, Lp-2002694p8, Lp-56207, 2735, H101460286, H101740836, H074360702, H134660746, H073300079, H073300077, H073360657, H092520167, H092620872, H103620682, H102860194, Lp-284, Lp-282-1, EUL 90, EUL 82, EUL 85, EUL 88, EUL 95, EUL 94, EUL 93, EUL 84, H085060063, H084800579, ID_2948, EUL 21, EUL 14, EUL 13, EUL 16, Lp-120, Lp-119, Lp-121, Lp-122, Lp-285, Lp-283, Lp-286-1, NIIB223, NIIB225, NIIB80, OLDA1, EUL 53, ID_1690, EUL 62, EUL 67, EUL 58, EUL 55, LP43, EUL 1, LT40-04, EUL 60, ID_891, ID_598, ID_2947, H124240908, H124600775, H103120165, H103340763, EUL 17, H072740379, EUL 109, EUL 104, LP22_Sweden, LP23_Sweden, LP21_Sweden, EUL 108, HL 0416 3014,Wien_47-14, HL 0036 4001, Paris, HL 0101 3003, HL 0102 3034, HL 0102 3035, Paris_2001_I_n2, HL 0051 1015, HL 0131 3039, LG 0713 5006, LG 0713 5007, EUL 3, EUL 10, EUL 9, LG 0940 4015, EUL 42, HL 0230 4015, LG 1020 3012, LG 1019 1002, HL 0311 1005, LG 1101 1012, LG 1105 4025, LG 0919 2006, HL 0337 3012, LG 0725 3019, LG 0725 3022, LG 1427 4010, LG 1427 4009, LG 0918 2002, LG 0918 2005, LG 1416 4007, LG 1416 4008, ID_1828, E21203, EUL 43, EUL 37, EUL 44, EUL 45, H104780628, H100120270, H100280679, H114840679, H114820438, H114840676, H114840681, H114840678, H114840680, H114840677, H072300480, H072300481, H100180615, H100180614, H100280685, H072360603, H111920394, H100560549, H100280683, H100280682, H100560548, H111920402, H100180616, H104720329, H112000588, H072680210, H100180617, H100120260, H104780627, H120680630, H072680211, H104780626, H111920400, H113440613, H113440616, H113440614, H113580550, H113440612, H113440615, H113580549, H072560534, H072680212, H072680213, H111920398, H072360604, H111920404, H115180236, H115260949, H091640624, H091720529, H100200320, H100200321, H100200319, H152780272, H152640286 |
| 1546535 | 1549931 | 3397 | EUL 157, Lp-032, L00-549, LG 1118 1044, EUL 119, EUL 114, EUL 113, LG 1014 3009, EUL 110, HL 0701 3004, ID_6885, ATCC 35289, EUL 46, LG 1139 1124, LP15, LP14, LP37, LP31, LP30, LP40, LP41, LP45, LP46, LP20, LP02, LP04, LP03, LP01, LP05, LP24, LP10, LP27, LP26, LP28, LP19, LP16, LP18, LP25, LP39, LP38, LP11, LP34, LP36, LP29, LP35, LP12, LP17, LP09, LP32, LP21, LP44, LP06, LP33, LP23, LP48, LP47, LP07, LP08, ID_2041, ID_1688, ID_747970, Lp-2002694p8, Lp-56207, 2735, H101460286, H101740836, H074360702, H134660746, H073300079, H073300077, H073360657, H092520167, H092620872, H103620682, H102860194, Lp-284, Lp-282-1, EUL 90, EUL 82, EUL 85, EUL 88, EUL 95, EUL 94, EUL 93, EUL 84, H085060063, H084800579, ID_2948, EUL 21, EUL 14, EUL 13, EUL 16, Lp-120, Lp-119, Lp-121, Lp-122, Lp-285, Lp-283, Lp-286-1, NIIB223, NIIB225, NIIB80, OLDA1, EUL 53, ID_1690, EUL 62, EUL 67, EUL 58, EUL 55, LP43, EUL 1, LT40-04, EUL 60, ID_891, ID_598, ID_2947, H124240908, H124600775, H103120165, H103340763, EUL 17, H072740379, EUL 109, EUL 104, LP22_Sweden, LP23_Sweden, LP21_Sweden, EUL 108, HL 0416 3014,Wien_47-14, HL 0036 4001, Paris, HL 0101 3003, HL 0102 3034, HL 0102 3035, Paris_2001_I_n2, HL 0051 1015, HL 0131 3039, LG 0713 5006, LG 0713 5007, EUL 3, EUL 10, EUL 9, LG 0940 4015, EUL 42, HL 0230 4015, LG 1020 3012, LG 1019 1002, HL 0311 1005, LG 1101 1012, LG 1105 4025, LG 0919 2006, HL 0337 3012, LG 0725 3019, LG 0725 3022, LG 1427 4010, LG 1427 4009, LG 0918 2002, LG 0918 2005, LG 1416 4007, LG 1416 4008, ID_1828, E21203, EUL 43, EUL 37, EUL 44, EUL 45, H104780628, H100120270, H100280679, H114840679, H114820438, H114840676, H114840681, H114840678, H114840680, H114840677, H072300480, H072300481, H100180615, H100180614, H100280685, H072360603, H111920394, H100560549, H100280683, H100280682, H100560548, H111920402, H100180616, H104720329, H112000588, H072680210, H100180617, H100120260, H104780627, H120680630, H072680211, H104780626, H111920400, H113440613, H113440616, H113440614, H113580550, H113440612, H113440615, H113580549, H072560534, H072680212, H072680213, H111920398, H072360604, H111920404, H115180236, H115260949, H091640624, H091720529, H100200320, H100200321, H100200319, H152780272, H152640286 |

**Figure S1.** A zoomed-in section of the maximum likelihood tree presented in Figure 1, showing environmental isolates from and clinical isolates linked to Hospital A, as well as closely-related isolates. Bootstrap values derived from 100 resamples are shown for each node.


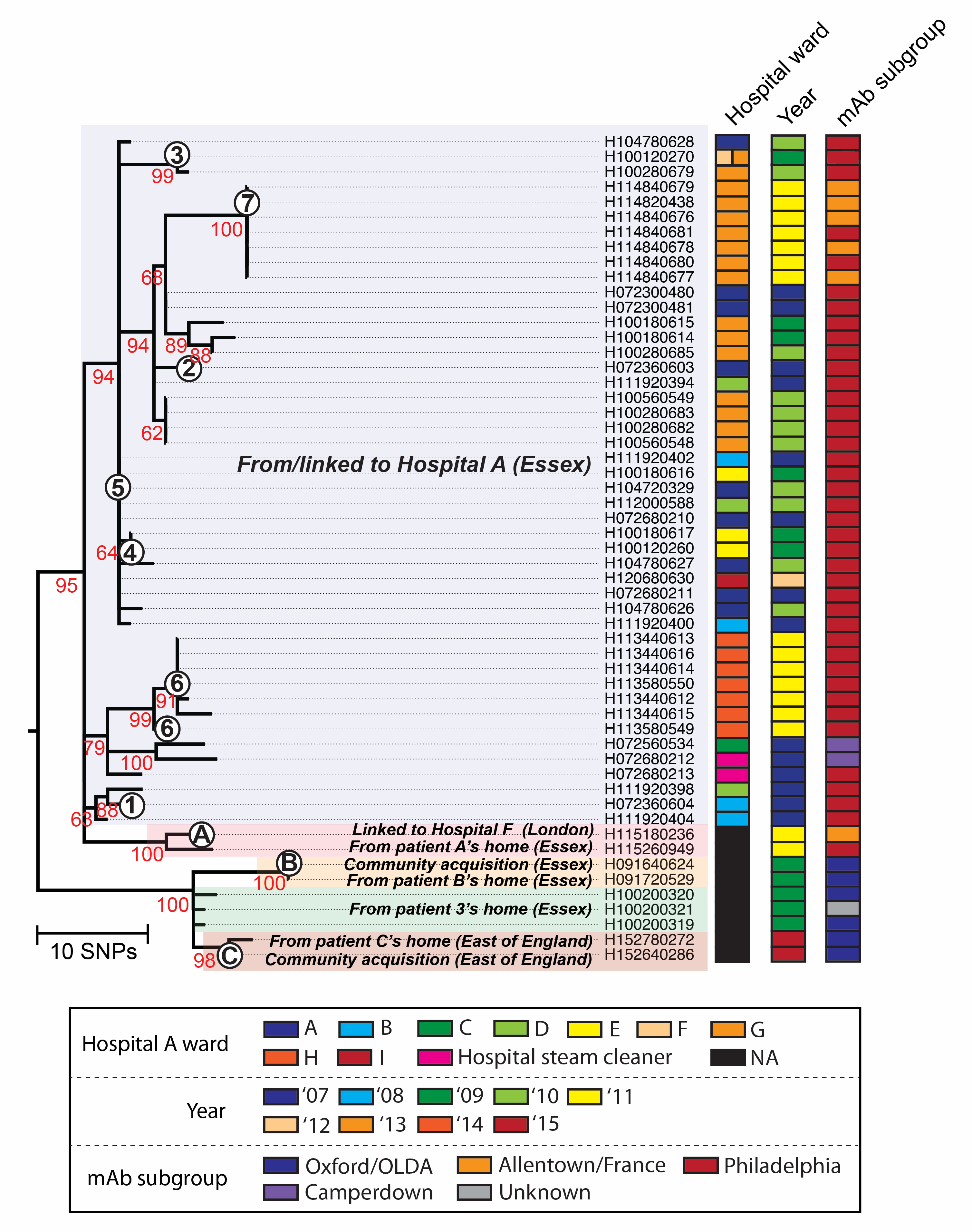


**Figure S2.** Proportion of combinations where clinical isolates associated with Hospital A are found to be derived from environmental isolates from Hospital A (i.e. good genomic evidence of hospital acquisition is achieved) when different numbers of environmental isolates are analysed. For each subset size of environmental isolates used, all possible combinations of the 38 isolates from Hospital A were used, up to a maximum of 100,000. A horizontal dotted line marks the level at which 90% of combinations achieve good genomic evidence of hospital acquisition for each subset size of environmental isolates analysed.


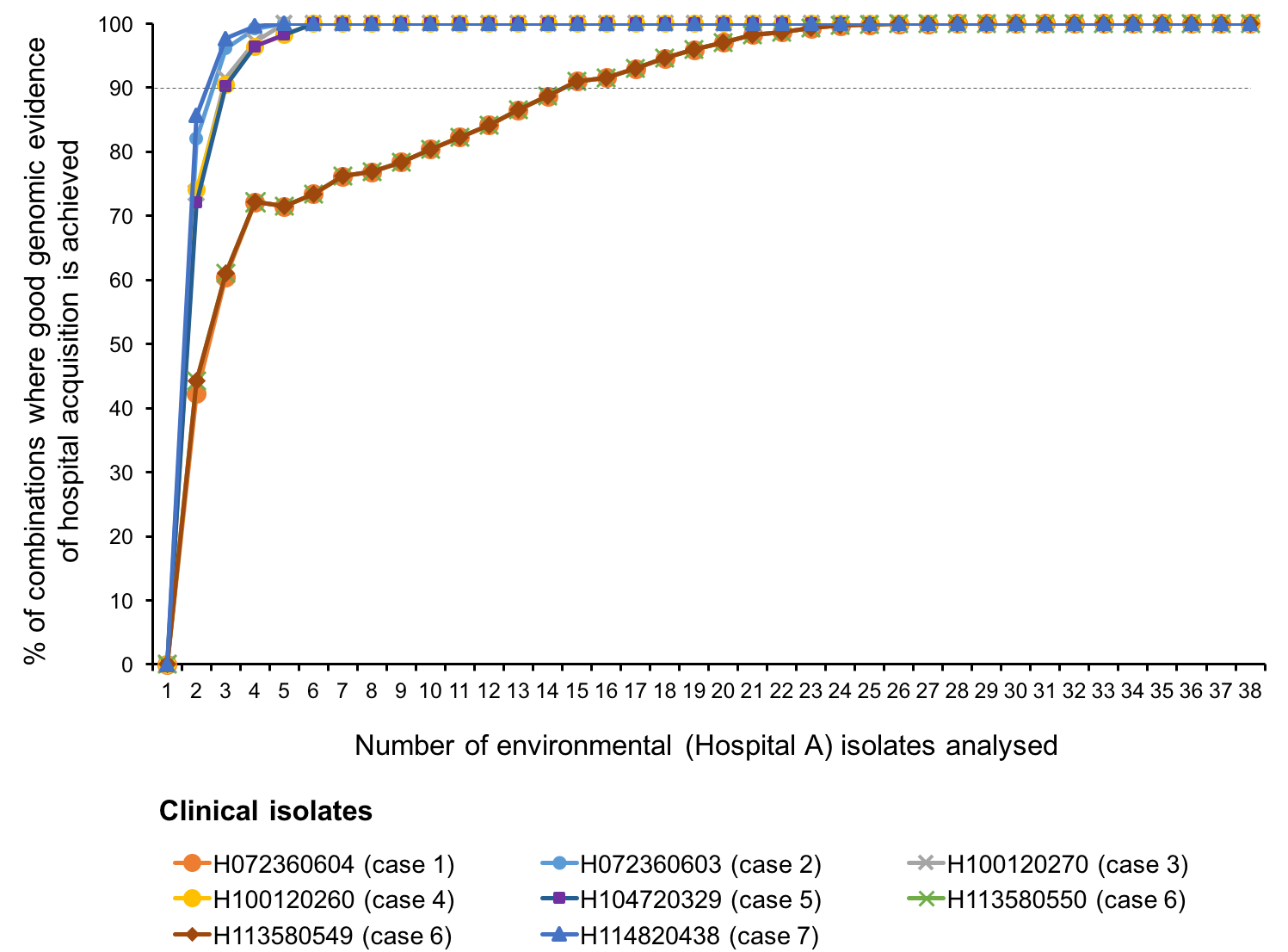

Supplement: David_2016_revised_supplementary_material [file cix153_suppl_David_2016_revised_supplementary_material.docx]
